# Transcriptional intermediary factor 1 gamma-based multitarget gene therapeutic strategy for triple-negative breast cancer

**DOI:** 10.1038/s41392-026-02977-x

**Published:** 2026-09-29

**Authors:** Hyomin Park, Tae Yoon Kim, Hyunji Yun, Areum Cha, Dodam Moon, Eugene Yun, Youngmin Kim, Seock-Ah Im, Dae-Won Lee, Changhee Park, Hong-Kyu Kim, Han-Byoel Lee, Jaewon Lee, Nam-Joon Cho, Eun Ju Lee, Hyo-Soo Kim

**Affiliations:** 1 Department of Molecular Medicine and Biopharmaceutical Sciences, Graduate School of Convergence Science and Technology, Seoul National University, Seoul, Republic of Korea; 2 Biomedical Research Institute, Seoul National University Hospital, Seoul, Republic of Korea; 3 Interdisciplinary Program in Stem Cell Biology, Seoul National University of Medicine, Seoul, Republic of Korea; 4 Cancer Research Institute, Seoul National University, Seoul, Republic of Korea; 5 Department of Internal Medicine, Seoul National University Hospital, Seoul National University College of Medicine, Seoul, Republic of Korea; 6 Department of Surgery, Seoul National University Hospital, Seoul National University College of Medicine, Seoul, Republic of Korea; 7 School of Materials Science and Engineering, Nanyang Technological University, Nanyang, Singapore; 8 Department of Biomedical Science, Seoul National University Hospital, Seoul, Republic of Korea

**Keywords:** Breast cancer, Gene therapy, Cancer microenvironment

## Abstract

Triple-negative breast cancer (TNBC) is an aggressive subtype lacking effective targeted therapies. A multitarget gene therapy was developed and validated to overcome molecular heterogeneity and compensatory survival signaling in TNBC. A codon-optimized human transcriptional intermediary factor 1 gamma (opti-hTIF1γ) gene therapy was evaluated in ex vivo–cultured patient biopsy tissues and in orthotopic and mammary intraductal mouse models, and the underlying mechanisms were investigated using pathway and immune-functional analyses. Ex vivo findings were further validated using patient-derived cells and complementary mechanistic studies, including ubiquitination assays, chromatin immunoprecipitation, and functional macrophage coculture analyses, to define the molecular basis of TIF1γ-mediated antitumor activity. Transduction of opti-hTIF1γ to ex vivo-cultured biopsy tissues from TNBC patients suppressed epithelial-to-mesenchymal transition and proliferation while inducing apoptosis. In orthotopic and mammary intraductal mouse models, opti-hTIF1γ effectively suppressed tumor growth and lung metastasis. Mechanistically, opti-hTIF1γ inhibits β-catenin via ubiquitination-dependent degradation and inhibits the SMAD-dependent TGFβ pathway by binding to SMAD2/3. In parallel, it suppresses the SMAD-independent TGFβ pathway via ubiquitination and caspase-3-associated degradation of STAT3, leading to the inhibition of TAK1. Furthermore, opti-hTIF1γ downregulates STAT3-dependent immune modulators such as CD47 and CXCL5 in TNBC, enhancing macrophage phagocytosis. These findings position opti-hTIF1γ as a promising multitarget gene therapeutic strategy for TNBC through concurrent suppression of tumorigenic signaling and reprogramming of the immune landscape.

## Introduction

Triple-negative breast cancer (TNBC), defined by the absence of estrogen receptor, progesterone receptor, and human epidermal growth factor receptor 2, accounts for ~15–20% of all breast cancers.^1^ TNBC is a clinically aggressive subtype characterized by early recurrence, visceral metastasis, and poor survival compared with other breast cancer subtypes.^2^ It occurs disproportionately in younger patients and exhibits marked intertumoral and intratumoral heterogeneity, complicating therapeutic stratification and limiting durable clinical responses.^2,3^ Despite advances in precision oncology and cancer immunotherapy, effective targeted treatment options for TNBC remain limited.^3^ Current treatment still relies largely on chemotherapy, whereas immune checkpoint inhibitors and selected targeted agents provide meaningful benefit only to molecularly defined patient subsets.^3^ Importantly, most targeted therapies inhibit individual signaling pathways or single molecular targets. Although inhibition of TGFβ, Wnt/β-catenin, or STAT3 has shown encouraging preclinical activity, clinical efficacy has frequently been limited by pathway redundancy, adaptive signaling rewiring, dose-limiting toxicity, and compensatory activation of alternative survival pathways.^4^ Consequently, TNBC readily escapes pathway-specific inhibition, leading to therapeutic resistance and disease progression. These limitations highlight the need for therapeutic strategies capable of simultaneously suppressing multiple interconnected oncogenic pathways rather than targeting individual signaling molecules.

Among the signaling networks driving TNBC progression, transforming growth factor-β (TGFβ) and β-catenin pathways play central roles in epithelial-to-mesenchymal transition (EMT), cancer stemness, invasion, metastasis, and therapeutic resistance.^5–8^ While TGFβ functions as a tumor suppressor during early tumorigenesis, persistent activation in advanced disease promotes EMT, immune suppression, extracellular matrix remodeling, and metastatic colonization.^5,7^ β-catenin, the principal effector of canonical Wnt signaling, similarly promotes proliferation, stemness, and EMT-associated transcriptional programs and cooperates with TGFβ through reciprocal crosstalk to reinforce malignant progression.^6–8^ Another key signaling hub is signal transducer and activator of transcription 3 (STAT3), a non-canonical mediator of TGFβ signaling that is constitutively activated in many cancers and regulates proliferation, invasion, inflammation, and immune evasion.^9–11^ In TNBC, STAT3 also links tumor-intrinsic signaling to the tumor microenvironment (TME) by regulating tumor-associated macrophages (TAMs). Rather than existing as fixed M1 or M2 populations, TAMs exhibit remarkable plasticity and are frequently reprogrammed by tumor-derived signals into tumor-supportive states that promote immune escape, angiogenesis, matrix remodeling, and metastatic dissemination.^12–15^ Together, these interconnected pathways establish a feed-forward network that drives tumor cell plasticity while creating an immunosuppressive microenvironment, indicating that durable therapeutic responses will likely require simultaneous modulation of both tumor-intrinsic signaling and the immune ecosystem.

This signaling complexity exposes a fundamental limitation of current TNBC therapies: inhibition of a single pathway rarely produces sustained responses because parallel and compensatory signaling networks remain active. A more effective strategy may therefore involve restoration of an upstream regulatory molecule capable of coordinately controlling multiple oncogenic pathways. Transcriptional intermediary factor 1 gamma (TIF1γ; also known as TRIM33) is a multifunctional protein that functions as both a transcriptional regulator and an E3 ubiquitin ligase.^16^ Unlike conventional targeted therapeutics directed against individual downstream effectors, TIF1γ occupies an upstream regulatory position where it modulates signaling through transcriptional regulation, chromatin remodeling, and ubiquitination-dependent protein degradation. Previous studies, including our own, identified TIF1γ as a critical negative regulator of TGFβ-SMAD signaling with potent antifibrotic activity.^17,18^ Emerging evidence further indicates that TIF1γ broadly suppresses oncogenic signaling by regulating the stability and transcriptional activity of multiple oncogenic regulators. Consistent with this concept, endogenous TIF1γ expression is frequently reduced in human cancers, whereas TGFβ, β-catenin, and STAT3 signaling are commonly activated, suggesting that loss of TIF1γ contributes to signaling imbalance and tumor progression.^19,20^ These observations raise the possibility that restoring TIF1γ activity could simultaneously suppress multiple oncogenic pathways while limiting compensatory signaling responses. Rather than inhibiting individual downstream targets, restoration of an endogenous master regulator may represent a distinct therapeutic paradigm for overcoming pathway redundancy, adaptive resistance, and immune evasion in TNBC.

In the present study, we investigated whether restoration of TIF1γ activity, achieved through delivery of a codon-optimized TIF1γ gene, could serve as a mechanistically defined multitarget therapeutic strategy for TNBC. We therefore investigated this strategy using a codon-optimized human TIF1γ construct (opti-hTIF1γ) and evaluated its therapeutic efficacy using complementary TNBC models, including mechanistic studies in cultured cells, ex vivo patient-derived biopsy tissues and primary cells, and orthotopic and mammary intraductal (MIND) mouse models. Ubiquitination assays, chromatin immunoprecipitation, and functional macrophage coculture analyses were further performed to define the molecular basis of TIF1γ-mediated antitumor activity. We demonstrate that opti-hTIF1γ coordinately suppresses multiple tumor-promoting pathways by inducing ubiquitination-dependent degradation of β-catenin, inhibiting canonical TGFβ signaling through interaction with SMAD2/3, and attenuating non-canonical TGFβ signaling via ubiquitination- and caspase-3-dependent degradation of STAT3, leading to inhibition of TAK1 signaling. In addition, opti-hTIF1γ reduces expression of the STAT3-regulated immune modulators CD47 and CXCL5, thereby enhancing macrophage-mediated antitumor activity and remodeling the immunosuppressive tumor microenvironment. Collectively, our findings identify TIF1γ restoration as a mechanistically defined multitarget therapeutic strategy that coordinately suppresses tumor-intrinsic oncogenic signaling and remodels the immune microenvironment through complementary molecular mechanisms. Beyond establishing opti-hTIF1γ as a promising gene therapy candidate for TNBC, this study provides a conceptual framework for treating aggressive cancers through restoration of an endogenous master regulator that coordinately controls multiple oncogenic and immune-associated signaling networks.

## Results

### Gene transfer of codon-optimized hTIF1γ into ex vivo-cultured biopsy tissues from TNBC patients suppresses EMT and proliferation while inducing apoptosis in cancer cells

**Fig. 1:**
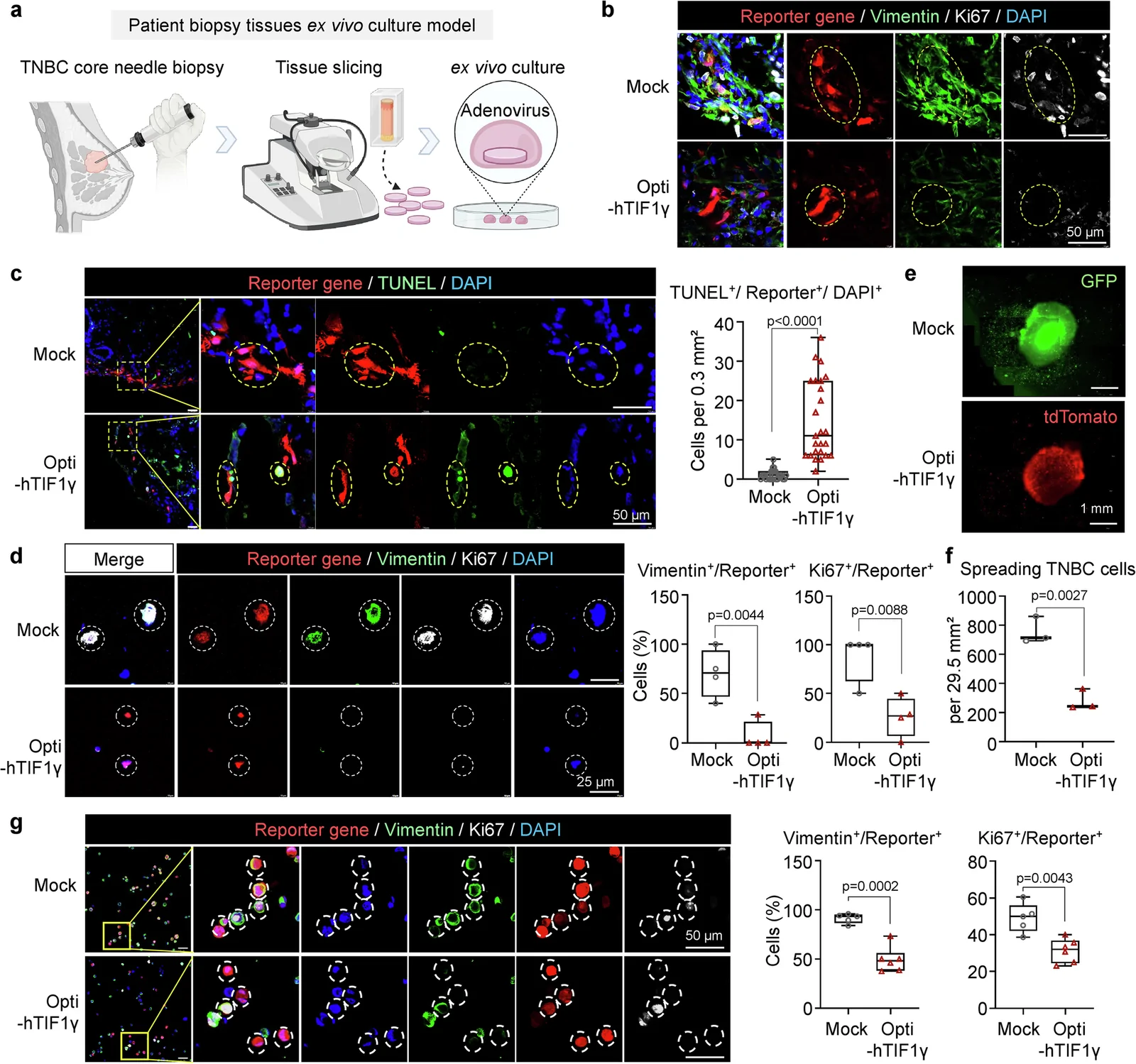
Codon-optimized hTIF1γ suppresses EMT and proliferation while inducing apoptosis in TNBC patient-derived tumor tissues. **a** Schematic overview of the ex vivo culture protocol. Illustration created with BioRender. **b** Immunofluorescence images of TNBC patient-derived tissue sections transduced with Ad-mock or Ad-opti-hTIF1γ-tdTomato, stained for DAPI (blue), a reporter gene (tdTomato or EGFP; pseudocolored red), vimentin (green), and Ki67 (white). Scale bars, 50 μm. **c** TUNEL assay of tissue sections showing increased apoptosis in the Ad-opti-hTIF1γ group. Triple-positive cells for DAPI, reporter gene (tdTomato), and TUNEL are marked with yellow dashed circles. Scale bars, 50 μm. Quantification of triple-positive (TUNEL^+^/reporter^+^/DAPI^+^) apoptotic cells per 0.3 mm^2^ area. Data represent the mean ± SD; quantification was performed for all patient-derived samples (P1–P7), *n* ≥ 3 for each sample. **d** Immunofluorescence spreading of TNBC cells from ex vivo-cultured TNBC biopsy slices, representing dissemination-associated phenotypes. DAPI (blue), a reporter gene (tdTomato or EGFP; pseudocolored red), vimentin (green), and Ki67 (white). Scale bars, 25 μm. Vimentin and Ki67 were used to assess EMT-associated and proliferative phenotypes, respectively, and were quantified as a percentage of the reporter-positive cell population per 0.78 mm^2^ field. Data represent the mean ± SD; *n* ≥ 3. **e** Representative images of disseminated TNBC cells from ex vivo-cultured mouse tumor tissues that were transduced with Ad-mock (GFP) or Ad-opti-hTIF1γ-tdTomato for 3 days. Scale bars, 1 mm. **f** Quantification of cells disseminated from tumor tissue per 29.5 mm^2^. Data represent the mean ± SD; *n* = 3. **g** Immunofluorescence images of cells disseminated from tumor tissue stained for DAPI, reporter gene (red), vimentin, and Ki67. Reporter-positive cells in the Ad-mock group expressed GFP (pseudocolored red) and were compared with Ad-opti-hTIF1γ-tdTomato cells. Scale bars, 50 μm. Quantification of vimentin^+^/reporter^+^ and Ki67^+^/reporter^+^ cells per 0.3 mm^2^ area. Data represent the mean ± SD; *n* = 5. Vimentin^+^/reporter^+^ cells; Ki67^+^/reporter^+^ cells

To enhance TIF1γ expression efficiency, the native TIF1γ sequence was codon-optimized and cloned into a plasmid (opti-hTIF1γ). To validate this optimization, both the codon-optimized plasmid and the wild-type TIF1γ plasmid were transiently transfected into cells. The codon-optimized construct yielded significantly higher protein production than the native sequence at equivalent transfection doses (Supplementary Fig. 1). To evaluate the antitumor and antimetastatic effects of opti-hTIF1γ, we established an ex vivo culture model using tumor biopsy tissues from patients with TNBC (P1–P7; Supplementary Table 1) and transduced the tissues with adenoviral vectors encoding opti-hTIF1γ. Supplementary Table 1 summarizes the molecular and clinicopathologic characteristics of the seven TNBC patient biopsy samples used in this study. Core needle biopsy samples were collected from seven patients. Biopsy tissues were sectioned and cultured ex vivo, followed by adenoviral transduction to induce opti-hTIF1γ expression (Fig. 1a). Cancer cells were transduced ex vivo with either a reporter-only construct (CMV-tdTomato, mock) or an opti-hTIF1γ construct linked to a fluorescent reporter (CMV promoter-driven opti-hTIF1γ/IRES-tdTomato). Reporter-positive cells were identified as successfully transduced. Immunofluorescence staining of tumor tissue sections from seven patients with TNBC (P1–P7) revealed that cancer cells transduced with opti-hTIF1γ exhibited markedly reduced levels of the EMT marker vimentin and the proliferation marker Ki67, indicating suppression of EMT and proliferation in patient-derived TNBC cells (Fig. 1b, Supplementary Fig. 2a).

Apoptosis in the P1–P7 tissues was assessed using the terminal deoxynucleotidyl transferase dUTP nick-end labeling (TUNEL) assay. While the control sample showed minimal TUNEL-positive cells, opti-hTIF1γ-transduced cells exhibited strong TUNEL signals, along with nuclear fragmentation. Triple colocalization analysis of nuclear staining, reporter gene (tdTomato), and TUNEL signals confirmed a significant increase in cell death upon opti-hTIF1γ introduction (Fig. 1c, Supplementary Fig. 2b).

To assess whether opti-hTIF1γ phenotypically suppresses tumor cells disseminated from the tumor mass, we analyzed spreading tumor cells that were detached from ex vivo-cultured biopsy slices into the culture supernatant. Compared to mock transduction, opti-hTIF1γ transduction significantly reduced the expression of both vimentin and Ki67 in these spreading TNBC cells (Fig. 1d).

To reproduce the patient-derived ex vivo culture system, we established a mouse tumor ex vivo culture using orthotopic TNBC tumors generated by injecting MDA-MB-231 human cells into the mammary fat pads of female mice. Tumor slices were transduced ex vivo with mock or opti-hTIF1γ adenovirus with fluorescent reporters for visualization and quantification of spreading tumor cells (Supplementary Fig. 3). In the mock-transduced group, the number of spreading tumor cells increased over 3 days, whereas opti-hTIF1γ transduction suppressed cell dissemination by ~70% (Fig. 1e, f). The spreading tumor cells were further analyzed using immunofluorescence staining. Cells transduced with opti-hTIF1γ exhibited lower expression of vimentin and Ki67 than those transduced with mock adenovirus (Fig. 1g).

### Orthotopic gene therapy using opti-hTIF1γ effectively suppresses primary tumor growth and lung metastasis in orthotopic TNBC mouse models

**Fig. 2:**
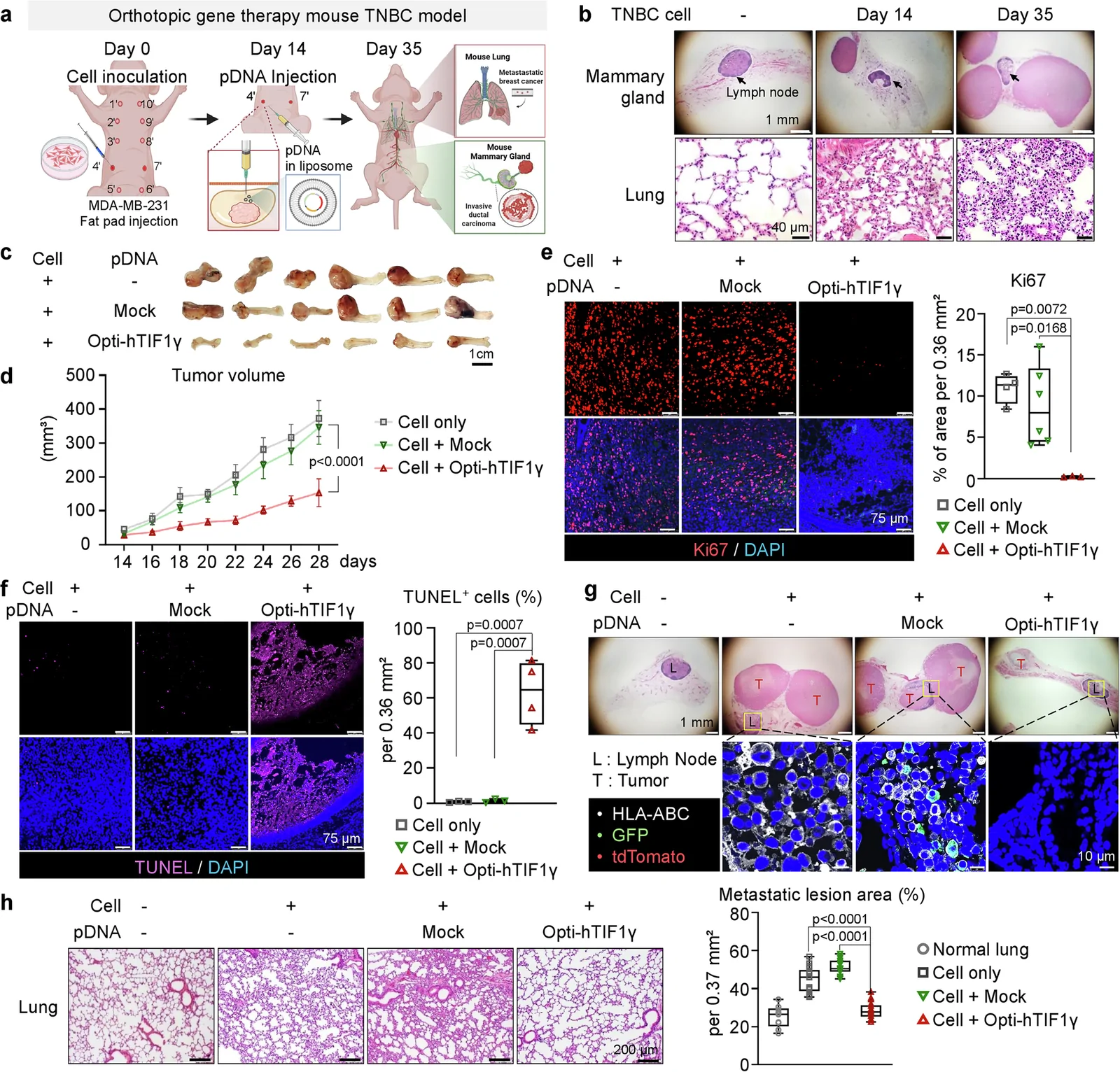
Orthotopic gene therapy using opti-hTIF1γ effectively suppresses primary and lung metastatic TNBC in mice. **a** Schemes of orthotopic gene therapy for primary and lung metastatic TNBC using MDA-MB-231 cells in mice. Illustration created with BioRender. **b** Representative H&E staining images of mammary gland and lung tissues at 14 and 35 days after orthotopic injection of TNBC (MDA-MB-231) cells, showing progressive tumor growth and pulmonary metastasis. Scale bars, 1 mm (top) and 40 μm (bottom). **c** Representative images of resected mammary glands on day 35. **d** Tumor growth measured from day 14 to 28. Tumor volume was calculated using the hemi-ellipsoid formula (0.5236 × length × width × height). Data represent the mean ± SEM; *n* = 6 tumors per group. **e** Immunofluorescence staining for Ki67 (red) and DAPI (blue) in tumor tissues. Scale bars, 75 μm. Quantification of Ki67^+^-positive % of area per 0.36 mm^2^. Data represent the mean ± SD; *n* ≥ 3 fields per group. **f** TUNEL staining (magenta) to assess apoptosis. Nuclei were counterstained with DAPI (blue). Scale bars, 75 μm. Quantification of TUNEL-positive % of area per 0.36 mm^2^. Data represent the mean ± SD; *n* ≥ 3 fields per group. **g** H&E images of mammary glands and immunofluorescence analysis of lymph nodes in the orthotopic TNBC model after mock (phMGFP) or opti-hTIF1γ (tdTomato) gene therapy. Human tumor cells were identified by HLA-ABC (white), and reporter genes were visualized as GFP (green) or tdTomato (red); nuclei were stained with DAPI (blue). Scale bars, 1 mm (H&E images) and 10 μm (IF images). **h** H&E staining of lung tissues collected from each group. Scale bars, 200 μm. Quantification of lung metastatic lesion % of area per field using ImageJ. Data represent the mean ± SD; *n* ≥ 3 fields per mouse, ≥3 mice per group

To evaluate the antitumor effects of opti-hTIF1γ in vivo, we established a mouse model of TNBC encompassing both primary tumor growth and lung metastasis. MDA-MB-231 human TNBC cells were injected into the mammary fat pad, and liposome-encapsulated mock (phMGFP) or opti-hTIF1γ (tdTomato) construct was injected into the peritumoral area within the mammary fat pad (Fig. 2a). Tumor burden in the mammary glands and lungs was evaluated using hematoxylin and eosin (H&E) staining on days 14 and 35 (Fig. 2b). At the endpoint, mice in the control groups (no injection or mock injection) had large tumors (>300 mm^3^), whereas those in the opti-hTIF1γ group exhibited significantly smaller or hardly observable tumors in the primary site (Fig. 2c, d).

Cell proliferation within tumor tissues was assessed using Ki67 immunofluorescence. Ki67-positive cells were abundant in the control groups, whereas they were markedly reduced in the opti-hTIF1γ group. The Ki67-positive cell area was reduced by >90% in the opti-hTIF1γ group compared to the control groups, indicating a significant antiproliferative effect of hTIF1γ (Fig. 2e). TUNEL-positive cell numbers were increased in opti-hTIF1γ-injected tumors versus control tumors, indicating enhanced cell death (Fig. 2f).

To evaluate lymph node metastasis, tumor cells were traced using human-specific human leukocyte antigen (HLA)-ABC immunofluorescence staining. In the plasmid control group, numerous HLA-ABC-positive cells, including reporter-positive cells, were detected in the lymph nodes. In contrast, in the opti-hTIF1γ group, neither tdTomato (reporter gene)-expressing cells nor other HLA-ABC-positive cells were observed. This finding suggested that tumor cells died before reaching the lymph nodes or that their migration ability was suppressed (Fig. 2g).

The effect of opti-hTIF1γ on lung metastasis was assessed via H&E staining of lung tissues. In the control groups, infiltrating tumor lesions were observed, and alveolar structures were severely disrupted. In contrast, lung tissues from the opti-hTIF1γ group showed normal alveolar structures without signs of tumor infiltration (Fig. 2h).

### Tumor cells transfected with opti-hTIF1γ show low metastatic activity from the mammary duct to the lungs in a mammary intraductal (MIND) TNBC mouse model

To assess the effect of opti-hTIF1γ on the lung metastasis of TNBC cells, a MIND (Mammary INtraDuct) model^21^ was established. MDA-MB-231 TNBC cells were pretransfected with either a phMGFP plasmid (mock cells) or an opti-hTIF1γ plasmid containing a tdTomato reporter (opti-hTIF1γ cells) and injected into the mammary ducts (Supplementary Fig. 4a). In H&E staining, control cells (wild-type or mock-transduced cells) produced numerous lung metastases by day 35, whereas opti-hTIF1γ-transduced cancer cells did not give rise to discernible lung metastatic lesions (Supplementary Fig. 4b).

Immunofluorescence staining for human-specific HLA-ABC confirmed the presence of human tumor cells in the lungs of mice injected with control cells but not in those injected with opti-hTIF1γ cells (Supplementary Fig. 4c). Notably, Ki67 immunofluorescence staining of lung tissues revealed numerous Ki67-positive (proliferative) tumor cells in the control groups, whereas lungs from mice injected with opti-hTIF1γ cells showed minimal proliferation and near-normal histology (Supplementary Fig. 4d).

### Opti-hTIF1γ suppresses tumorigenic traits in TNBC cells by regulating proliferation, apoptosis, and EMT

**Fig. 3:**
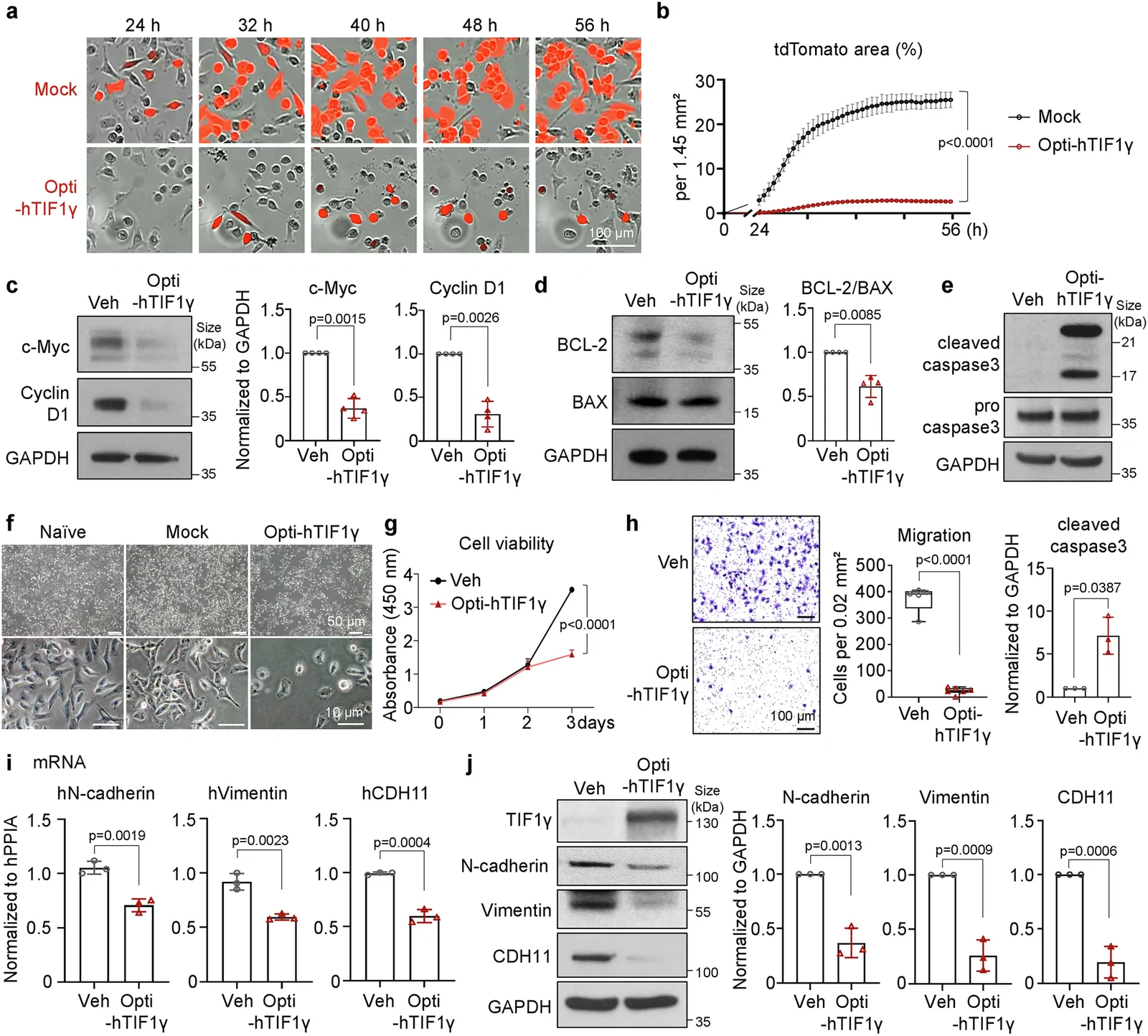
Opti-hTIF1γ reduces tumorigenic traits by regulating proliferation, apoptosis, and EMT in TNBC cells. **a** Representative time-lapse images of MDA-MB-231 cells transfected with control-tdTomato or opti-hTIF1γ-tdTomato plasmid, captured from 24 to 56 h posttransfection. Red indicates tdTomato expression. Scale bars, 100 μm. **b** Quantification of tdTomato intensity over time. Data represent the mean ± SD; *n* ≥ 3 wells per group, with ≥ 4 fields analyzed per well. **c**–**e** Western blot analysis of tumorigenesis and cell cycle progression-associated proteins (c-Myc and Cyclin D1) and apoptosis-related proteins (BCL-2, BAX, cleaved caspase-3 and total caspase-3) in transfected MDA-MB-231 cells. GAPDH was used as a loading control. Protein expression in **c** and **e** was densitometrically quantified relative to GAPDH, whereas in **d**, apoptosis-related changes were assessed by calculating the BCL-2/BAX ratio. Data represent the mean ± SD; *n* = 3. **f** Representative phase-contrast images of MDA-MB-231 cells 48 h after transfection with mock or opti-hTIF1γ plasmids, as well as wild-type cells. Scale bars, 50 μm (top) and 10 μm (bottom). **g** Cell Counting Kit-8 assay showing cell viability over 3 days after plasmid transfection. The absorbance at 450 nm was measured daily. Data represent the mean ± SD; *n* = 3 replicates. **h** Migration of MDA-MB-231 cells in the Transwell assay stained with crystal violet. Scale bars, 100 μm. Quantification of migrating cells per 0.02 mm^2^. Data represent the mean ± SD; *n* ≥ 5 fields per group. **i** RT‒qPCR analysis of EMT-associated genes (N-cadherin, vimentin, and CDH11) in transfected MDA-MB-231 cells. Data are normalized to hPPIA expression and presented as the mean ± SD; *n* = 3. **j** Western blot analysis of TIF1γ and EMT markers (N-cadherin, vimentin, and CDH11) normalized to GAPDH. Densitometric quantitative data represent the mean ± SD; *n* = 3

To further investigate the antitumor effects of opti-hTIF1γ observed in human samples and animal models, its effects on cell proliferation, apoptosis, and related gene expression were evaluated using TNBC cell lines. MDA-MB-231 cells were transfected with a tdTomato plasmid (mock) or an opti-hTIF1γ plasmid containing tdTomato (opti-hTIF1γ) and analyzed using live-cell imaging (24–56 h posttransfection). In the mock group, tdTomato-positive cell numbers continuously increased, whereas in the opti-hTIF1γ group, tdTomato-positive cells did not increase in number and showed apoptotic features, including shrinkage and fragmentation (Fig. 3a, b, Supplementary Videos 1, 2).

Consistent with these phenotypes, opti-hTIF1γ reduced the expression of oncogenic proteins associated with tumorigenesis and cell cycle progression (c-Myc and Cyclin D1) (Fig. 3c) and altered the expression of apoptosis-related proteins (Fig. 3d, e). The BCL-2/BAX ratio was significantly decreased, primarily due to a reduction in the antiapoptotic BCL-2 rather than an increase in BAX (Fig. 3d). The cleaved caspase-3 level was increased, indicating the induction of apoptosis (Fig. 3e). Compared with cells in the wild-type and mock groups, cells in the opti-hTIF1γ group exhibited significant inhibition of proliferation, along with a distinctive apoptotic morphology (Fig. 3f). Furthermore, viability assays showed that opti-hTIF1γ significantly suppressed cell proliferation (Fig. 3g).

We next examined the mechanistic impact of hTIF1γ on EMT and motility in MDA-MB-231 cells. Cells in the opti-hTIF1γ group showed marked reductions in both cell migration (Fig. 3h) and EMT-associated gene expression at the mRNA and protein levels (Fig. 3i, j).

### Opti-hTIF1γ suppresses β-catenin and TGFβ signaling via transcriptional and posttranslational mechanisms in TNBC cells

**Fig. 4:**
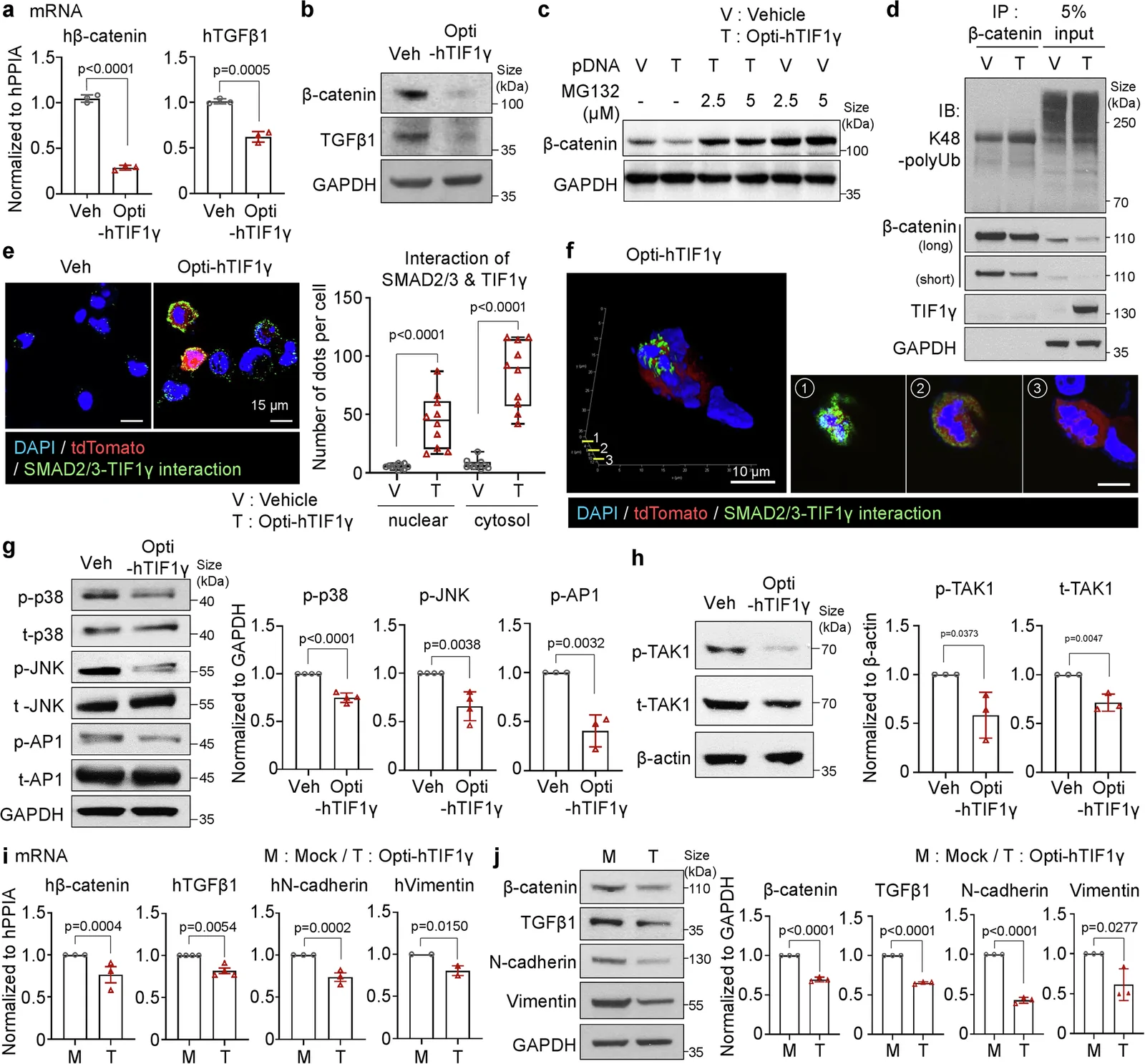
Opti-hTIF1γ suppresses β-catenin and TGFβ signaling via transcriptional and posttranslational mechanisms. **a** RT‒qPCR analysis in MDA-MB-231 cells transfected with mock or opti-hTIF1γ plasmid. Data are normalized to hPPIA expression and presented as the mean ± SD; *n* = 3. **b** Western blot analysis in MDA-MB-231 cells. GAPDH was used as a loading control. **c** Western blot analysis of β-catenin in TNBC cells transfected with vehicle control or TIF1γ and treated with MG132 (2.5 and 5 μM). GAPDH served as a loading control. **d** IP of β-catenin followed by immunoblotting for K48-linked polyubiquitin in MDA-MB-231 cells. **e** PLA images showing interactions between TIF1γ and SMAD2/3 (green) in MDA-MB-231 cells, with the tdTomato reporter gene (red) and DAPI staining for nuclei (blue). Scale bars, 15 μm. Quantification of PLA puncta per cell in nuclear and cytosolic compartments. Data represent the mean ± SD; *n* ≥ 10 cells per group. **f** Representative Z-stack image of an opti-hTIF1γ-transfected cell from (**e**). Images ①–③ show individual optical sections at different Z-depths. Scale bars, 10 μm. **g** Western blot analysis of SMAD-independent signaling protein (p38, JNK, and AP1) phosphorylation and expression in MDA-MB-231 cells. Densitometric quantification was normalized to GAPDH. Data represent the mean ± SD; *n* ≥ 3. **h** Western blot analysis of phosphorylated and total TAK1 levels in MDA-MB-231 cells. β-Actin was used as a loading control. Data represent the mean ± SD; *n* = 3. **i** RT‒qPCR analysis of β-catenin, TGFβ and EMT-associated genes in opti-hTIF1γ-transduced PDCs. Data were normalized to hPPIA expression and represent the mean ± SEM; independent experiments, with each spot representing a distinct experiment and including at least duplicate measurements. **j** Western blot analysis of β-catenin, TGFβ, and EMT markers in opti-hTIF1γ-transduced PDCs. GAPDH was used as a loading control. Data represent the mean ± SD; *n* = 3

Small interfering RNA (siRNA) knockdown of β-catenin and TGFβ was used to validate their functional roles in TNBC cell proliferation and migration. Consistently, siRNA-mediated suppression of β-catenin and TGFβ led to suppressed cell proliferation and migration (Supplementary Fig. 5a, b). Transduction of opti-hTIF1γ significantly reduced the mRNA and protein levels of β-catenin and TGFβ in TNBC cells (Fig. 4a, b, Supplementary Fig. 5c).

We next aimed to elucidate the mechanisms by which opti-hTIF1γ inhibits the above signaling pathways. Consistent with the known role of TIF1γ in promoting β-catenin degradation via K48-linked ubiquitination,^22^ MG132 partially rescued β-catenin protein levels in TIF1γ-overexpressing cells, and IP analysis showed increased K48-linked polyubiquitination of β-catenin, indicating enhanced ubiquitination-dependent degradation (Fig. 4c, d, Supplementary Fig. 5d).

The role of opti-hTIF1γ in the two major TGFβ pathways (SMAD-dependent and SMAD-independent) was also investigated. In the SMAD-dependent pathway, SMAD2/3 form a complex with SMAD4 to promote downstream signaling. Proximity ligation assays (PLAs) performed after opti-hTIF1γ transduction in MDA-MB-231 cells revealed an enhanced interaction between opti-hTIF1γ and SMAD2/3 (Fig. 4e). This was accompanied by nuclear fragmentation, suggesting apoptosis due to SMAD pathway inhibition (Fig. 4f).

In the SMAD-independent pathway, opti-hTIF1γ reduced the phosphorylation levels of the key downstream mediators p38, JNK, and AP1 (Fig. 4g). Given that these kinases function downstream of TGFβ-activated kinase 1 (TAK1), we therefore examined phosphorylated and total TAK1 levels, which decreased upon opti-hTIF1γ transfection, highlighting TAK1 as a critical target of opti-hTIF1γ in this signaling cascade (Fig. 4h). Consistent with the data obtained with the TNBC cell line, patient‑derived cells (PDCs) also showed reduced β‑catenin, TGF‑β, and EMT marker expression upon opti‑hTIF1γ transduction (Fig. 4i, j).

### Opti-hTIF1γ inhibits the SMAD-independent TGFβ-activated kinase 1 (TAK1) pathway through ubiquitination and caspase-3-associated degradation of STAT3

**Fig. 5:**
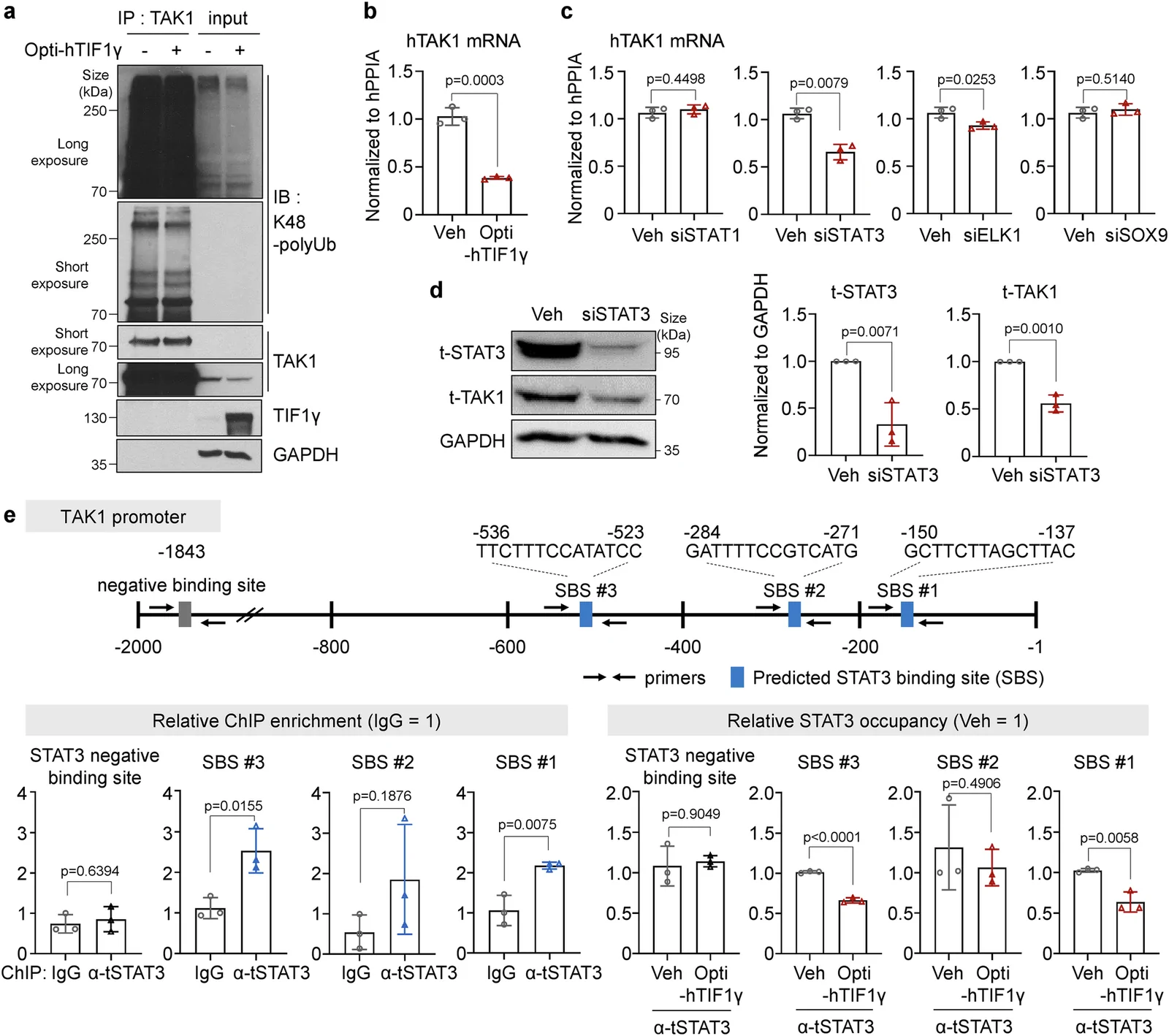
hTIF1γ downregulates TAK1 expression through STAT3-dependent transcriptional repression. All data (**a**–**e**) were obtained using MDA-MB-231 cells. Data represent the mean ± SD. *n* = 3. **a** Co-IP analysis of TAK1 in MDA-MB-231 cells with or without opti-hTIF1γ transfection. Cell lysates were immunoprecipitated with anti-TAK1 and immunoblotted for K48-linked polyubiquitin, TAK1, and TIF1γ. GAPDH was used as a loading control. **b** RT‒qPCR analysis of TAK1 mRNA levels in cells transfected with mock or opti-hTIF1γ plasmid. Data represent the mean ± SD; *n* = 3. **c** RT‒qPCR analysis of TAK1 expression following siRNA-mediated knockdown of candidate transcription factors. Data represent the mean ± SD; *n* = 3. **d** Western blot and densitometric quantification of STAT3 and TAK1 protein levels following siSTAT3 treatment. Data were normalized to GAPDH. Data represent the mean ± SD; *n* = 3. **e** (Top) Schematic representation of the TAK1 (MAP3K7) promoter region showing the primer locations for the negative control region and the three predicted STAT3-binding sites (SBSs) located at −536 to −523 (SBS #3), −284 to −271 (SBS #2), and −150 to −137 (SBS #1) relative to the transcription start site. (Bottom) ChIP‒qPCR analysis of STAT3 enrichment at the negative control region and predicted STAT3-binding sites, normalized to IgG (IgG = 1), and relative STAT3 occupancy in cells transfected with vehicle control (Veh) or opti-hTIF1γ normalized to vehicle (Veh = 1). Data represent the mean ± SD; *n* = 3

Given the pronounced reductions in both phosphorylated and total TAK1 protein levels after opti-hTIF1γ transduction (Fig. 4h), we investigated how opti-hTIF1γ inhibits TAK1 activation. TAK1 is a key SMAD-independent TGFβ pathway mediator that promotes tumor progression.^23,24^ To determine whether opti-hTIF1γ inhibits TAK1 via its E3 ligase activity, IP assays were conducted to assess K48-linked polyubiquitination. Although total TAK1 levels were reduced in opti-hTIF1γ-transduced cells, K48-linked ubiquitination of TAK1 remained unchanged, suggesting that TAK1 degradation is not driven by the enzymatic activity of opti-hTIF1γ (Fig. 5a). Instead, TAK1 mRNA levels were significantly reduced, indicating transcriptional regulation (Fig. 5b).

**Fig. 6:**
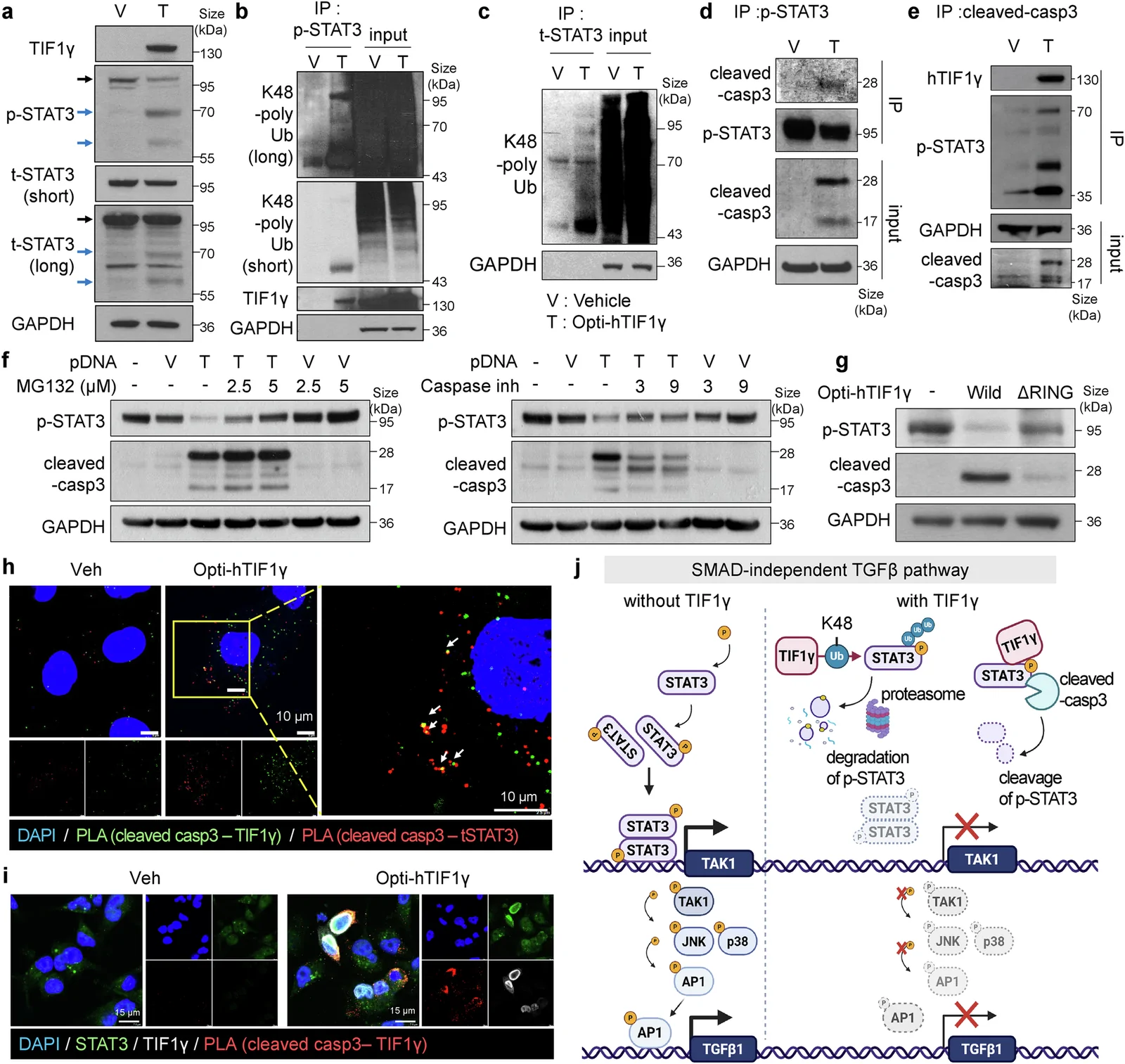
TIF1γ promotes dual degradation of p-STAT3 (Y705) through E3 ligase-dependent ubiquitination and caspase-3-mediated cleavage, suppressing SMAD-independent TGFβ signaling in TNBC. **a** Western blot analysis of Y705-p-STAT3, total-STAT3, and STAT3 degradation products in mock- or opti-hTIF1γ-transfected cells. Black arrows indicate full-length STAT3, whereas blue arrows indicate STAT3 fragments. **b** Co-IP of Y705-p-STAT3, followed by immunoblotting with K48-linked polyubiquitin and TIF1γ. **c** Co-IP of total STAT3, followed by immunoblotting with K48-linked polyubiquitin. **d** Co-IP of Y705-p-STAT3, followed by immunoblotting with cleaved caspase-3. **e** Co-IP of cleaved caspase-3, followed by immunoblotting with TIF1γ and Y705-p-STAT3. **f** Left, Western blot analysis of pSTAT3 (Y705) and cleaved caspase-3 in TNBC cells transfected with vehicle control or TIF1γ expression construct and treated with MG132 at the indicated concentrations (2.5 and 5 μM). Right, Western blot analysis of pSTAT3 (Y705) and cleaved caspase-3 in TNBC cells transfected with vehicle control or TIF1γ and treated with caspase-3 (Z-DEVD-FMK) or caspase-9 inhibitors (Z-LEHD-FMK) (20 μM). GAPDH was used as a loading control. **g** Western blot analysis of p-STAT3 in cells transfected with wild-type TIF1γ or the RING domain mutant (ΔRING: C125A and C128A). **h** Representative PLA images. Blue: DAPI (nuclei); green: PLA signals indicating interactions between cleaved caspase-3 and TIF1γ; red: PLA signals indicating interactions between cleaved caspase-3 and total STAT3. Right: Magnified view (yellow box) shows colocalization of green and red PLA signals (white arrows). Scale bars, 10 μm. **i** Dual immunofluorescence (IF) and PLA showing interactions and expression patterns of cleaved caspase-3, STAT3, and TIF1γ. PLA (red) indicates cleaved caspase-3–TIF1γ interactions; IF signals show total STAT3 (green) and TIF1γ (white). Nuclei are counterstained with DAPI (blue). Scale bars, 15 μm. **j** Schematic illustration of opti-hTIF1γ-mediated p-STAT3 degradation via ubiquitination and caspase-3. Illustration created with BioRender

To identify the transcription factor responsible for this downregulation, in silico transcription factor-binding site prediction was performed using the MAP3K7 (TAK1) promoter region, yielding four candidate regulators: STAT1, STAT3, ELK1, and SOX9. SiRNA-mediated knockdown studies of these candidates revealed that *STAT3* knockdown had the strongest suppressive effect on TAK1 mRNA expression (Fig. 5c). siSTAT3 reduced TAK1 protein levels, confirming STAT3-dependent TAK1 regulation (Fig. 5d). As STAT3 activation depends on Tyr705 phosphorylation, which facilitates dimerization and nuclear translocation,^25^ we next examined whether TIF1γ alters STAT3 occupancy at the MAP3K7 promoter. ChIP‒qPCR analysis showed that TIF1γ overexpression significantly reduced STAT3 binding at the predicted promoter sites, particularly SBS #3 and SBS #1, thereby supporting the direct transcriptional regulation of TAK1 (Fig. 5e). We next assessed p-STAT3 levels. Western blotting revealed a significant reduction in the level of full-length Tyr705-p-STAT3 upon opti-hTIF1γ transduction, accompanied by increased levels of lower-molecular-weight p-STAT3 fragments, suggestive of proteolytic degradation (Fig. 6a, Supplementary Fig. 6a). STAT3 mRNA expression remained unchanged (Supplementary Fig. 6b), supporting the notion that opti-hTIF1γ suppresses STAT3 posttranslationally. Nuclear/cytosol fractionation analysis revealed that the degradation products of p-STAT3 predominantly accumulated in the cytosolic fraction (Supplementary Fig. 6c).

Studies have suggested that STAT3 degradation is regulated via posttranslational mechanisms such as ubiquitination and caspase-mediated cleavage.^26–28^ Notably, opti-hTIF1γ enhanced K48-linked polyubiquitination of p-STAT3 (Fig. 6b) as well as total STAT3 (Fig. 6c), suggesting that opti-hTIF1γ degrades STAT3 via E3 ligase activity. Furthermore, co-IP analysis revealed that opti-hTIF1γ binds directly with p-STAT3 for ubiquitination (Fig. 6b).

In addition to ubiquitin-mediated degradation, cytosolic p-STAT3 fragmentation upon opti-hTIF1γ overexpression involved caspase-dependent mechanisms. Cleaved caspase-3 associated with p-STAT3 in opti-hTIF1γ-expressing cells (Fig. 6d), and in co-IP experiments, cleaved caspase-3 pulled down both opti-hTIF1γ and p-STAT3 fragments (Fig. 6e). Consistently, MG132 partially restored pSTAT3 levels, whereas caspase-3 inhibition reduced pSTAT3, indicating that proteasomal and caspase-dependent processes both contribute to pSTAT3 downregulation (Fig. 6f). To determine whether the E3 ligase activity of TIF1γ is required for p-STAT3 degradation, we compared WT-TIF1γ with a ΔRING mutant lacking E3 ligase activity. Unlike WT-TIF1γ, the ΔRING mutant failed to reduce p-STAT3 levels or induce cleaved caspase-3, indicating that intact E3 ligase activity is essential for both caspase-3 activation and p-STAT3 degradation (Fig. 6g, Supplementary Fig. 6d).

PLA and immunofluorescence showed enhanced interactions between cleaved caspase-3 and TIF1γ and between cleaved caspase-3 and STAT3 in the cytoplasm of opti-hTIF1γ-expressing cells, supporting the formation of a triple complex (TIF1γ/p-STAT3/cleaved caspase-3). In contrast, TIF1γ–STAT3 colocalization in PLA-negative regions suggested proteasomal degradation of STAT3 independent of caspase-3, with both pathways potentially acting concurrently or sequentially (Fig. 6h, i).

Opti-hTIF1γ suppressed the SMAD-independent TGFβ pathway by downregulating Y705-p-STAT3 at the protein level—through proteasomal degradation mediated by K48-linked ubiquitination and caspase-mediated cleavage—leading to reduced TAK1 expression and downstream signaling (Fig. 6j). Collectively, these findings suggested that opti-hTIF1γ simultaneously suppresses three major oncogenic signaling pathways—β-catenin, TGFβ/SMAD, and STAT3—through transcriptional inhibition, direct interaction, and protein degradation (Supplementary Fig. 6e).

### Opti-hTIF1γ gene therapy improves the TME of TNBC by facilitating cancer cell phagocytosis by TAMs

**Fig. 7:**
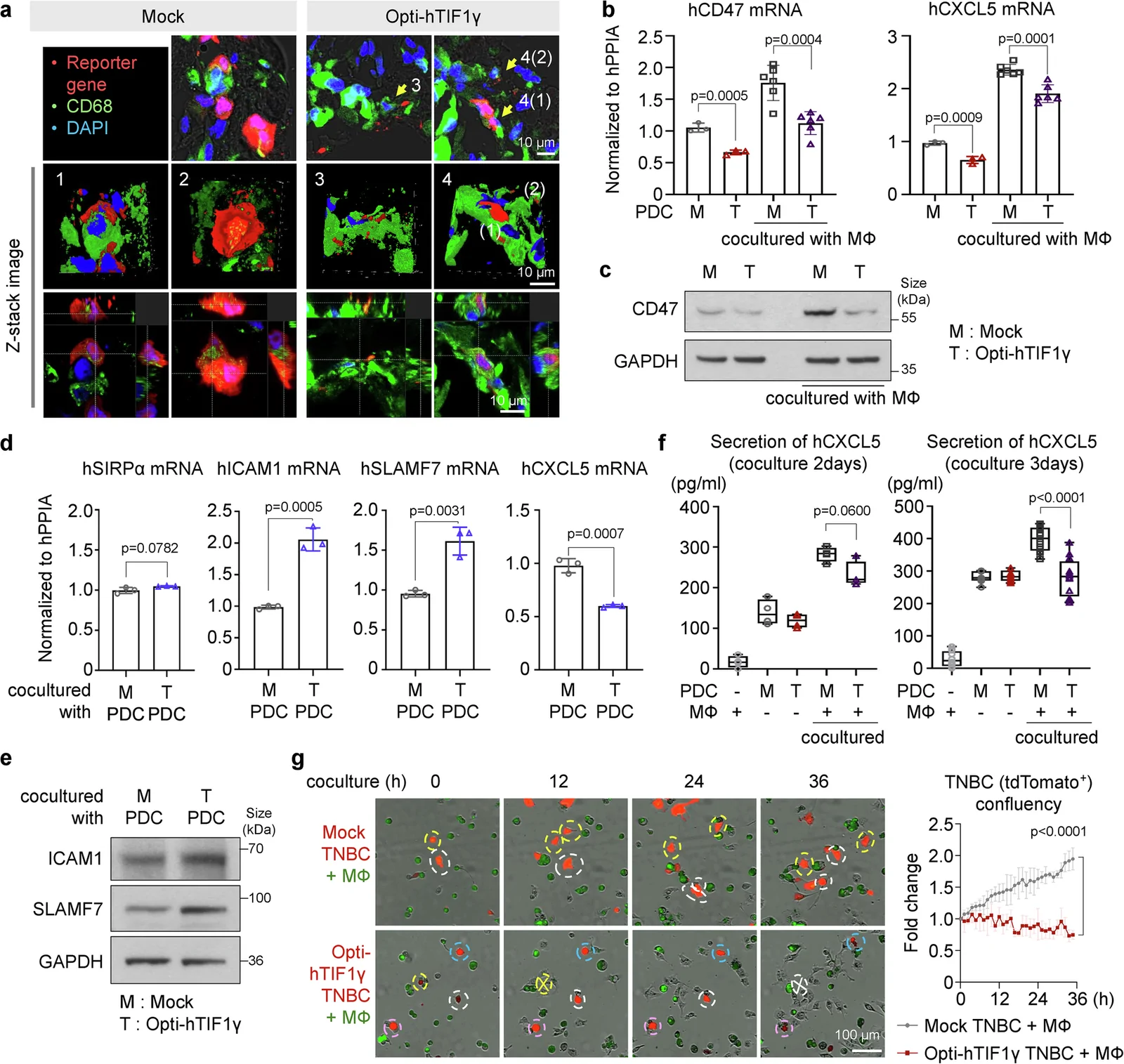
Opti-hTIF1γ downregulates the CD47–CXCL5 axis by inhibiting STAT3 in TNBC cells, leading to enhanced phagocytosis by macrophages. **a** Immunofluorescence staining of patient-derived TNBC tissues showing reporter-positive TNBC cells (red), CD68^+^ macrophages (green), and DAPI (blue). Z-stack confocal images illustrate the localization of TNBC cells outside (mock) or inside (opti-hTIF1γ) macrophages. Scale bars, 10 μm. **b** RT‒qPCR of CD47 and CXCL5 expression in PDCs with or without PDC-macrophage 2-layer coculture. Data represent the mean ± SD; *n* ≥ 3. **c** Western blot analysis of CD47 in PDCs with or without 2-layer coculture. **d** RT‒qPCR analysis of SIRPα, ICAM1, SLAMF7, and CXCL5 in THP-1 macrophages cocultured with PDCs. Data represent the mean ± SD; *n* = 3. **e** Western blot of ICAM1 and SLAMF7 in THP-1 macrophages cocultured with PDCs. **f** ELISA of CXCL5 levels in coculture supernatants on days 2 and 3. Data represent the mean ± SD; *n* ≥ 3. **g** Time-lapse live-cell imaging of THP-1 macrophages (green, DiI stained) cocultured with transfected MDA-MB-231 cells (red, reporter gene: tdTomato). Each dashed circle indicates the tracked morphology of individual TNBC cells. “X” in the opti-hTIF1γ group denotes the death of the tracked cell. Time-course quantification of cancer cell confluency. Scale bars, 100 μm. Confluency was measured using the instrument’s built-in software tool based on the area covered by tdTomato-positive cells per field. Values were normalized to the confluency at the starting time point (0 h) and are presented as fold change. Data represent the mean ± SEM; *n* = 3 wells, ≥ 2 fields per well

TAMs crosstalk with cancer cells within the tumor-supportive microenvironment.^29^ In ex vivo TNBC biopsy tissue cultures, mock-transduced cancer cells contacting macrophages (Figs. 7a-1) maintained an aggressive cancer morphology (Figs. 7a-2). In contrast, opti-hTIF1γ-transduced cancer cells appeared fragmented within CD68^+^ macrophages (Figs. 7a-3,4), suggesting active phagocytosis.

To elucidate the underlying mechanism, we examined the expression of CD47, a “don’t eat me” signal for macrophage-mediated phagocytosis evasion,^30^ and C-X-C motif chemokine ligand 5 (CXCL5), related to TAM–cancer cell crosstalk, within the TME.^31,32^ Opti-hTIF1γ decreased CD47 and CXCL5 gene and protein expression in PDCs (Supplementary Fig. 7a, b, c).

In a 2-layer coculture system of adenovirus-transduced PDCs and THP-1 macrophages induced with phorbol 12-myristate 13-acetate (PMA), opti-hTIF1γ-transduced PDCs consistently exhibited reduced CD47 and CXCL5 expression at both the mRNA and protein levels compared with mock-transduced PDCs (Fig. 7b, c, Supplementary Fig. 7d). Coculture with macrophages upregulated CD47 and CXCL5 expression in TNBC cells, but this macrophage-driven induction was suppressed by opti-hTIF1γ, indicating that opti-hTIF1γ interferes with macrophage-mediated protumoral signaling.

Conversely, in macrophages cocultured with opti-hTIF1γ-transduced TNBC PDCs, the expression of SIRPα, the cognate receptor for CD47, remained largely unchanged (Fig. 7d). In contrast, macrophages exposed to opti-hTIF1γ-expressing TNBC cells exhibited a distinct activation phenotype characterized by increased expression of SLAMF7, a receptor that promotes prophagocytic (“eat-me”) signaling, and ICAM1 (CD54), a marker of macrophage activation, along with a marked reduction in CXCL5 (Fig. 7d, e, f, Supplementary Fig. 7e).

To assess the effect of opti-hTIF1γ on cancer cell phagocytosis by macrophages, we cocultured TNBC cells (MDA-MB-231) and PMA-induced THP-1 macrophages (Supplementary Fig. 7f). Immunofluorescence staining to visualize phagocytic interactions revealed morphological differences between the groups (Supplementary Fig. 7g, h). Mock-transduced tdTomato^+^ cancer cells maintained an intact morphology even when in contact with ICAM1^+^ macrophages. In contrast, opti-hTIF1γ-transduced tdTomato^+^ cancer cells were fragmented or engulfed by ICAM1^+^ macrophages, indicating active phagocytosis. The phagocytosis rate was significantly increased in the opti-hTIF1γ group compared to the control group (Supplementary Fig. 7h). Live-cell imaging showed that while mock-transfected cancer cells proliferated normally, opti-hTIF1γ-expressing cells underwent apoptosis or were engulfed by macrophages (Fig. 7g, Supplementary Videos 3,4). In opti-hTIF1γ-transfected TNBC cells, CD47 and CXCL5 levels were significantly suppressed (Supplementary Fig. 7i, j). Even under TNFα stimulation, CXCL5 secretion from TNBC cells was reduced in the opti-hTIF1γ group (Supplementary Fig. 7k). Under coculture conditions, opti-hTIF1γ-transfected cancer cells were actively phagocytosed by macrophages, whereas mock-transfected cancer cells retained CD47 expression and were not efficiently phagocytosed (Supplementary Fig. 7l).

Noting the concurrent changes in CD47 and CXCL5 expression induced by opti-hTIF1γ, we performed an in silico promoter analysis of these genes, which identified putative STAT3-binding sites within the proximal promoter regions of both CD47 and CXCL5, suggesting that they are direct transcriptional targets of STAT3 (Supplementary Fig. 8a). To determine whether the effect of opti-hTIF1γ on these two genes was mediated via STAT3, we knocked down STAT3 using siRNA. STAT3 knockdown resulted in significant downregulation of CD47 and CXCL5 at both the mRNA and protein levels (Supplementary Fig. 8b, c, d). Together with the finding that opti-hTIF1γ suppressed STAT3 activation, these results suggested that the reductions in CD47 and CXCL5 in cancer cells induced by opti-hTIF1γ were mediated by STAT3 inhibition (Supplementary Fig. 8e).

**Fig. 8:**
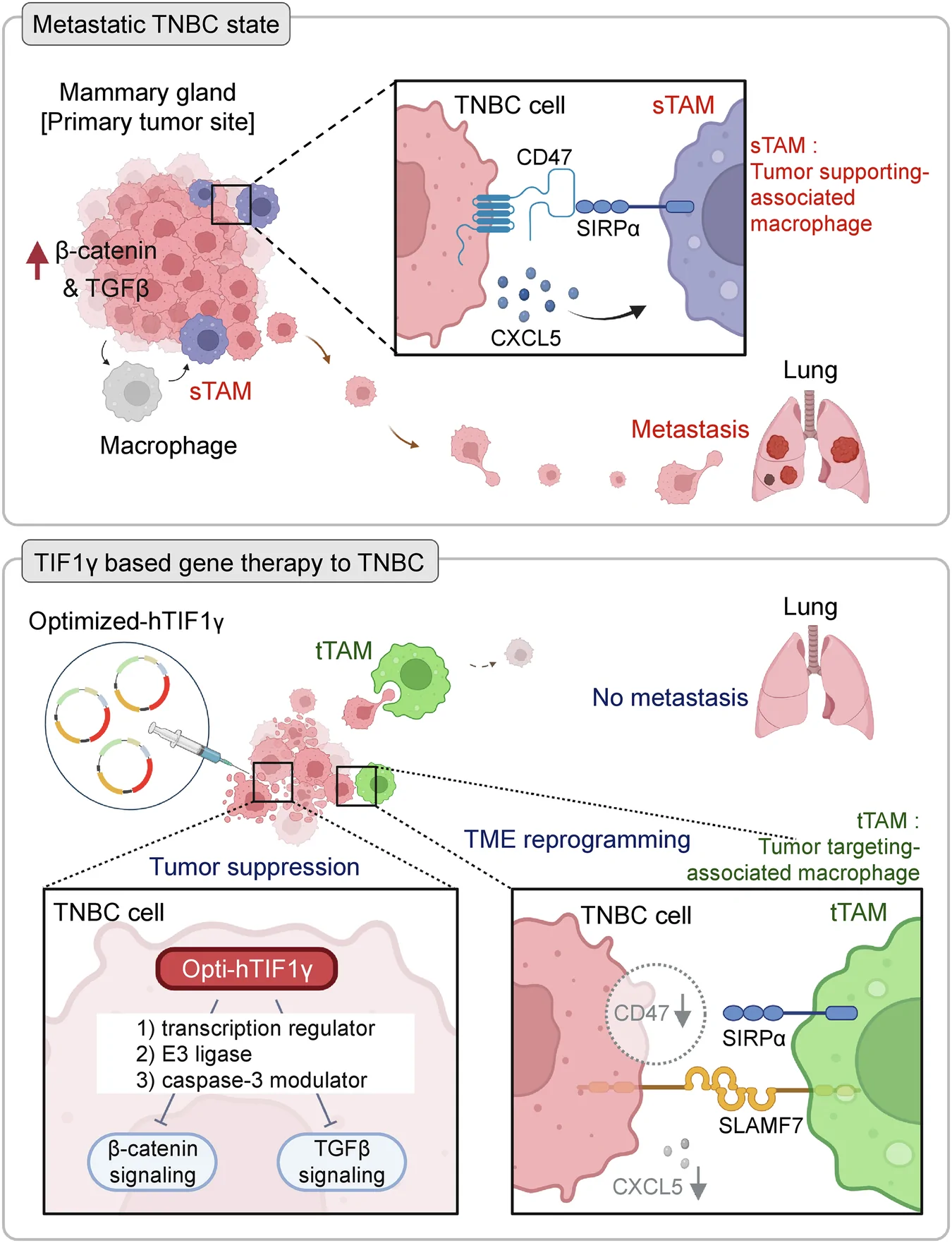
Schematic illustration of opti-hTIF1γ-mediated tumor suppression and TME reprogramming in TNBC. Schematic summary illustrating how opti-hTIF1γ suppresses tumorigenic and metastatic signaling in TNBC cells and reprograms TAMs toward a phagocytic, tumor-targeting phenotype. Illustration created with BioRender

Taken together, the results suggested that opti-hTIF1γ exerts dual antitumor effects: 1) it directly suppresses β-catenin and TGFβ signaling in TNBC cells, thereby inhibiting EMT and proliferation as well as enhancing cell death, and 2) it modulates the TME by downregulating CXCL5 and CD47 in TNBC cells and upregulating SLAMF7 in macrophages, promoting the transition of sTAMs to tumor-targeting TAMs (tTAMs) (Fig. 8).

## Discussion

We demonstrated the therapeutic efficacy of opti-hTIF1γ in inducing apoptosis and suppressing cell proliferation and EMT in TNBC using patient- and mouse-derived tumor biopsy tissues while reducing β-catenin and TGFβ expression at both the mRNA and protein levels. These findings are corroborated by previous findings that hTIF1γ suppresses β-catenin and TGFβ signaling in various cancer cell types.^18,22^ Notably, TIF1γ also functions as an E3 ubiquitin ligase that promotes β-catenin degradation. The partial, dose-dependent rescue of β-catenin protein by MG132 supports a primary role for K48-linked ubiquitination and proteasomal degradation.^22^ The accompanying reduction in β-catenin mRNA likely reflects indirect transcriptional consequences of reduced β-catenin/TCF signaling. However, direct transcriptional repression of β-catenin cannot be excluded, as in silico analysis identified putative SMAD-binding elements within the β-catenin (CTNNB1) promoter (Supplementary Fig. 9a), suggesting that TIF1γ may also influence β-catenin expression through SMAD-associated promoter regulation.

TIF1γ regulates both SMAD-dependent and SMAD-independent TGFβ signaling. For the SMAD-dependent pathway, we previously showed in hepatic stellate cells that TIF1γ interacted with SMAD2/3, preventing their association with SMAD4 and downstream signaling.^18^ In our present study, we demonstrated a similar interaction between TIF1γ and SMAD2/3 in TNBC cells, leading to apoptosis through inhibition of the SMAD-dependent TGFβ pathway. Our findings suggest that TIF1γ exerts additional non-SMAD effects, including pSTAT3 degradation and TAK1 regulation. Taken together, these data support the view that TIF1γ exerts context-specific antitumor effects in TNBC that extend beyond canonical SMAD regulation alone.

Because our initial analyses focused on canonical TGFβ-SMAD signaling, we extended our investigation to noncanonical pathways when canonical signaling alone could not fully account for the observed phenotype. The reduction in pSTAT3 without changes in STAT3 mRNA further suggested posttranslational regulatory mechanisms. Notably, the inability of the ΔRING mutant to suppress pSTAT3 indicates that intact E3 ubiquitin ligase activity is required for STAT3 regulation, supporting a predominant role for ubiquitin-dependent degradation rather than caspase-3-associated processing alone. Collectively, these findings support an interconnected mechanistic framework linking TIF1γ-mediated ubiquitination, STAT3 suppression, and downstream TAK1 regulation. Further studies will be required to define the contributions of individual TIF1γ domains and clarify the molecular basis of these regulatory interactions.

Regarding the SMAD-independent TGFβ pathway, we identified a previously unrecognized mechanism in which TIF1γ decreased the STAT3 protein level via ubiquitination and degradation, leading to TAK1 downregulation. Although little is known about total TAK1 regulation, TAK1 phosphorylation has been implicated in cancer cell survival.^33^ TIF1γ overexpression reduced TAK1 expression at both the mRNA and protein levels. Additionally, we identified STAT3 as a potential upstream regulator of TAK1 expression. STAT3 is known to be regulated posttranslationally via ubiquitination and caspase-mediated cleavage.^26–28^ Consistent with this mechanism, p-STAT3 and total STAT3 levels decreased in TIF1γ-overexpressing cells, whereas degradation products of p-STAT3 accumulated. Co-IP assays supported an interaction between TIF1γ and p-STAT3, accompanied by increased K48-linked ubiquitination of p-STAT3 and co-IP of cleaved caspase-3 and p-STAT3, suggesting complex formation. The observed decrease in STAT3 binding to the MAP3K7 promoter following TIF1γ overexpression supports a direct role for the TIF1γ–STAT3 axis in TAK1 regulation. However, our data do not exclude the involvement of additional cooperative or parallel pathways. Thus, TAK1 downregulation may reflect a multifactorial regulatory process in which STAT3 suppression represents an important, but not exclusive, component.

Based on these findings, we propose that TIF1γ promotes dual degradation of p-STAT3. Under basal conditions, TNBC cells exhibit high levels of p-STAT3, which suppresses caspase-3 activation and supports antiapoptotic signaling.^34^ Upon TIF1γ overexpression, p-STAT3 undergoes K48-linked polyubiquitination and proteasomal degradation, impairing its dimerization and nuclear translocation. This likely relieves p-STAT3-mediated inhibition of caspase-3 cleavage. Furthermore, TIF1γ (or a TIF1γ/p-STAT3 complex) may recruit cleaved caspase-3 and stabilize the complex, promoting further proteolytic cleavage of p-STAT3 and suppressing downstream tumorigenic signaling. This cascade results in enhanced caspase-3 activation and apoptotic cell death. Thus, TIF1γ may suppress STAT3 signaling through both its E3 ligase activity and caspase-3-mediated processing. Further studies are warranted to elucidate the precise mechanism by which TIF1γ recognizes and regulates p-STAT3 stability.

Given its multitarget mode of action, opti-hTIF1γ may impose a higher barrier to resistance than single-pathway therapies by concurrently modulating β-catenin, TGFβ, and STAT3 signaling. However, we acknowledge that TNBC cells may still acquire adaptive escape mechanisms over time, including compensatory activation of parallel signaling or metabolic rewiring. Accordingly, future studies will be needed to define the durability of this response and to evaluate whether combination strategies may further reduce the likelihood of resistance.

TAMs exhibit functional plasticity and can be reprogrammed by the TME into tumor-supportive “sTAMs.” We therefore examined macrophage-mediated cancer cell phagocytosis through “eat me” and “don’t eat me” signals.^12,15,29,30^ Coculture with opti-hTIF1γ-transfected TNBC cells restored macrophage phagocytic activity toward cancer cells, and we referred to such reactivated macrophages as “tumor-targeting TAMs.” In ex vivo-cultured tumor tissues from TNBC patients, tdTomato-labeled cancer cells transduced with opti-hTIF1γ were frequently detected within CD68+ macrophages, indicating active cancer cell phagocytosis by TAMs.

Mechanistically, opti-hTIF1γ transfection led to reduced CD47 and CXCL5 expression in TNBC cells, contributing to enhanced phagocytosis by macrophages. CD47, a “don’t eat me” signal, prevents macrophage-mediated tumor cell clearance. Its downregulation in hTIF1γ-expressing TNBC cells likely enhanced macrophage phagocytosis, as highlighted by the increases in SLAMF7 (an “eat me” signal receptor) and ICAM1 (a marker of activation) expression in macrophages by opti-hTIF1γ. In parallel, CXCL5, a chemokine implicated in the recruitment of immunosuppressive macrophages and the formation of a tumor-supportive microenvironment,^31^ was downregulated in TNBC cells at both the mRNA and protein levels by TIF1γ. Notably, both CD47 and CXCL5 are transcriptionally regulated by STAT3,^35,36^ and our data support that TIF1γ suppresses STAT3 signaling, leading to the suppression of these immunomodulatory factors. TIF1γ is a known transcriptional coregulator, and its ability to modulate gene expression at the chromatin level should be considered. In silico analysis of the CD47 and CXCL5 promoter regions identified canonical SMAD-binding elements near predicted STAT3 response sites (Supplementary Fig. 9b). These findings suggest that, in addition to suppressing pSTAT3-dependent signaling, TIF1γ may also downregulate CD47 and CXCL5 expression through suppression of SMAD-associated transcriptional activation. Additional studies will be needed to clarify the relative contribution of these pathways.

From a clinical translation perspective, the route of administration and delivery efficiency are key considerations for metastatic disease. In our model, peritumoral administration of the liposomal vector was associated with reduced lung metastasis, which was well corroborated by the finding that reporter expression was observed in the mammary ducts and regional lymph nodes, suggesting access of therapeutic genes to local drainage pathways. Nevertheless, localized injection has clear limitations for widely disseminated disease, and systemic or tumor-targeted delivery platforms will be needed for broader clinical application. Our long-term toxicity data (Supplementary Fig. 10) indicate that repeated intraperitoneal administration of opti-hTIF1γ was well tolerated in mice, as no obvious histopathological abnormalities were detected in major organs, and serum chemistry remained largely unchanged after 41 weeks of treatment. These results support the initial safety profile of the liposomal delivery formulation. However, additional studies are warranted to further characterize biodistribution, tissue persistence, and potential off-target effects under clinically relevant dosing conditions.

When developing immune-modulating gene therapies, the intrinsic immunogenicity of the delivery vehicle should be carefully considered. Here, adenoviral vectors were used only for ex vivo transduction, whereas in vivo studies were conducted with a liposome-encapsulated plasmid system, which helps separate the observed therapeutic effects from adenoviral immunogenicity. Moreover, adoption of the mock adenovirus control in ex vivo assays supports the conclusion that the effects on CD47/CXCL5 and phagocytosis are linked to differences in TIF1γ expression rather than to the effect of the adenoviral backbone. Future studies will be needed to further reduce potential vector-associated innate immune priming through nonviral or stealth-engineered delivery systems.

For TIF1γ-based TNBC gene therapy, achieving sufficient intracellular expression is likely important for therapeutic activity. In this context, codon optimization may be useful for improving translational efficiency and increasing protein output from the therapeutic construct. However, efficient clinical translation will also require delivery strategies that improve tumor selectivity while minimizing off-target expression in normal tissues. Future approaches may benefit from tumor-responsive promoters, ligand-targeted nanoparticles, or other delivery systems that improve tumor-selective TIF1γ expression.

In conclusion, using human and mouse TNBC models, we demonstrated that opti-hTIF1γ gene therapy effectively inhibits the β-catenin and TGFβ pathways, reprograms tumorigenic and immune-related signaling, and exerts multifaceted antitumor effects. Furthermore, we identified a novel mechanism by which opti-hTIF1γ directly inhibits Y705-p-STAT3 activity to suppress CD47 and CXCL5, leading to the restoration of tumor-targeting TAMs. Collectively, these findings support restoration of endogenous TIF1γ as a multitarget therapeutic strategy for TNBC that concurrently suppresses tumor-intrinsic oncogenic signaling and remodels the immune microenvironment.

## Materials and methods

### Ethics approval

#### Human study

Tumor tissues were obtained from seven patients with TNBC treated at Seoul National University Hospital, Republic of Korea. All patients provided written informed consent for tissue donation. The study was approved by the Institutional Review Board of Seoul National University Hospital (IRB No. H-2307-101-1449).

#### Animal study

All animal experiments were conducted in accordance with the ARRIVE 2.0 (Animal Research: Reporting of In Vivo Experiments) guidelines.^37^ The protocols were approved by the Institutional Animal Care and Use Committee of Seoul National University Hospital, Republic of Korea (IACUC No. 20-0057-S1A1(2)).

### Cell culture

MDA-MB-231 TNBC cells and THP-1 human monocytes were obtained from the American Type Culture Collection (ATCC; Cat. Nos. HTB-26 and TIB-202, respectively). PDCs were isolated by culturing biopsy tissues from patients with TNBC ex vivo. Freshly obtained tumor specimens were mechanically dissociated into single cells and cultured ex vivo under adherent conditions identical to those used for MDA-MB-231 cells. To induce differentiation into macrophages, THP-1 cells were treated with 100 nM PMA for 24 h. All cells were maintained in RPMI 1640 medium (Cat. No. 11875093, Gibco) supplemented with 10% fetal bovine serum (FBS; Cat. No. F0900-050, GenDepot) and 1% penicillin‒streptomycin (P/S)-glutamine (Cat. No. 10378016, Gibco) in a humidified incubator at 37 °C with 5% CO_2_.

### Ex vivo culture of tumor tissues from TNBC patients and model mice

Fresh patient-derived tumor specimens were incubated in RPMI 1640 medium supplemented with 1% P/S for at least 2 h. Tissues were blotted to remove excess moisture and embedded in 1.5% low melting point agarose (Cat. No. 16520-100, Invitrogen). Serial 300 μm-thick sections were prepared using a vibratome (Leica VT1200s). Tissue sections were infected with adenovirus vectors (CMV-EGFP or CMV-opti-hTIF1γ-IRES-EGFP/tdTomato) by applying a 150 μL droplet of culture medium containing 5 × 10^9^ virus particles directly onto the surface of each slice and incubating them for 24 h. After transduction, the medium was replaced with RPMI 1640 supplemented with 10% FBS and 1% P/S. During ex vivo culture, disseminated TNBC cells spontaneously detached from the tissue slices. These cells were collected from the culture supernatant and subjected to immunofluorescence staining to evaluate migratory or proliferative behavior.

For fixation, the sections were fixed in 4% paraformaldehyde at room temperature ( ~ 25 °C) for 15 min. After fixation, the sections were washed with PBS and embedded in optimal cutting temperature (OCT) compound.

For the mouse ex vivo model, female BALB/c-nude mice were orthotopically injected in the mammary fat pad with 5 × 10^6^ MDA-MB-231 cells suspended in 0.04% trypan blue in PBS. Fourteen days after injection, tumor masses were excised, sliced into 300 μm sections, and subjected to ex vivo culture and adenoviral transduction using the same protocol as for human tissues. Disseminated cells released from the tumor slices during culture were visualized by fluorescence microscopy and collected for further analysis.

### Orthotopic TNBC mouse model

Female BALB/c-nude mice (5 weeks old, 16–20 g, Orient Bio) were used for all in vivo experiments. For orthotopic gene therapy of primary and lung metastatic TNBC in mice, 3 × 10^6^ MDA-MB-231 cells were suspended in 0.04% trypan blue in PBS and injected into the 4th or 7th mammary fat pad to induce primary tumors. Fourteen days post-injection, after confirming the primary tumor as well as lung metastasis, 18 µg of plasmid DNA encapsulated in 140 nmol of liposomes (Luca AICell) was injected orthotopically into the peritumoral area within the mammary fat pad. Tumor volume was measured using calipers and calculated using the hemi-ellipsoid formula (volume = 0.5236 × length × width × height).

### Live-cell imaging

For single-cell imaging of cancer cells, MDA-MB-231 cells were seeded in six-well plates and transfected with mock plasmids (encoding tdTomato only) or opti-hTIF1γ plasmids linked to a tdTomato reporter (Macrogen). At 24 h posttransfection, time-lapse live-cell imaging was initiated using a Celloger Pro system (Curiosis). Bright-field and red fluorescence channels (tdTomato) were captured every 10 min for a total of 32 h (endpoint: 56 h posttransfection).

For mixedcoculture imaging, MDA-MB-231 cells were transfected with mock or opti-hTIF1γ plasmids expressing tdTomato for 48 h. THP-1 monocytes were labeled with the dye DiO for 15 min. Transfected cancer cells were added to DiO-labeled macrophages at a 1:4 cancer cell:macrophage ratio in six-well plates. Live-cell imaging was initiated at the start of coculture and conducted for 36 h, capturing bright-field, green (DiO), and red (tdTomato) fluorescence images at 15 min intervals using the Celloger Pro system.

In both single and coculture imaging experiments, tdTomato fluorescence intensity (% area) was quantified using the software provided by the manufacturer.

### Co-IP

For co-IP experiments, MDA-MB-231 cells were lysed in ice-cold lysis buffer containing 1% Triton X-100 and 0.1% SDS and supplemented with protease and phosphatase inhibitors on ice for 15 min. The lysates were centrifuged at 15,000 × *g* and 4 °C for 10 min to collect the supernatant. A portion of the lysate (5%) was reserved as an input control. For IP, 2 µg of primary antibody was added to 1.5 mg of total protein and incubated on a rotator at 4 °C overnight. Immune complexes were captured via incubation with protein A/G agarose beads (Cat. No. ab196232, Abcam) at 4 °C under rotation for 2 h. Nonspecifically bound proteins were removed by washing with buffer containing 0.2% Tween-20. Subsequent immunoblotting was performed as described for western blot analysis.

### Using primers and antibodies in this study is listed supplementary tables 2–7

#### Statistical analysis

Statistical analyses were performed using GraphPad Prism 8 software (GraphPad Software, La Jolla, CA, USA). Statistical tests were chosen based on the data distribution and experimental design. For comparisons between two groups, the unpaired two-tailed t test or two-way ANOVA was used as appropriate. For comparisons involving more than two groups, one-way or two-way ANOVA was applied, followed by Sidak’s multiple comparisons test. Statistical significance was set to *p* < 0.05.

## Supplementary information

Supplementary information

The original uncropped western blot images

Video S1. Live imaging of mock-transfected TNBC cells

Video S2. Live imaging of opti-hTIF1γ–transfected TNBC cells

Video S3. Live imaging of mock-transfected TNBC cells cocultured with macrophages

Video S4. Live imaging of opti-hTIF1γ–transfected TNBC cells cocultured with macrophages

## Data Availability

All data supporting the findings of this study are provided within the article and its Supplementary Information files.
