# Supplementary material for: Transcriptional intermediary factor 1 gamma-based multitarget gene therapeutic strategy for triple-negative breast cancer
Source: Signal Transduct Target Ther. 2026 Sep 29;11:413. doi: 10.1038/s41392-026-02977-x (PMC13620165; doi:10.1038/s41392-026-02977-x)

Fig 3c

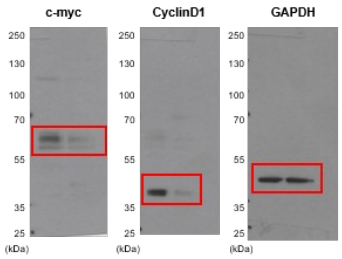

Fig 3d

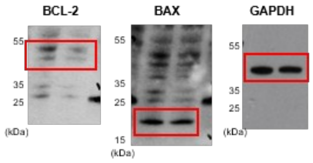

Fig 3e

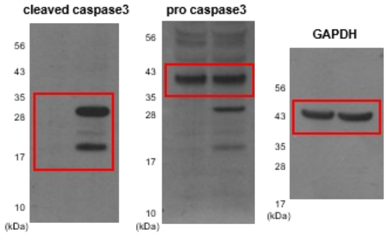

Fig 3j

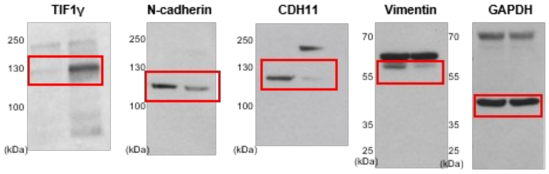

Fig 4b

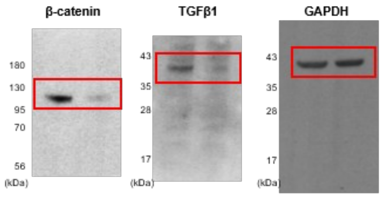

Fig 4c

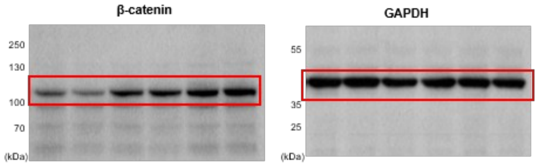

Fig 4d

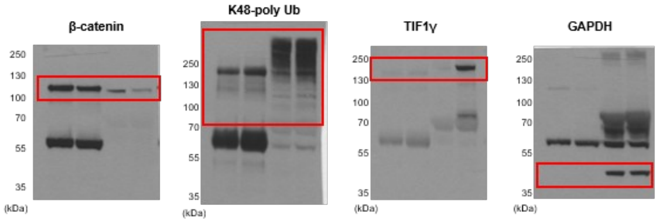

Fig 4g

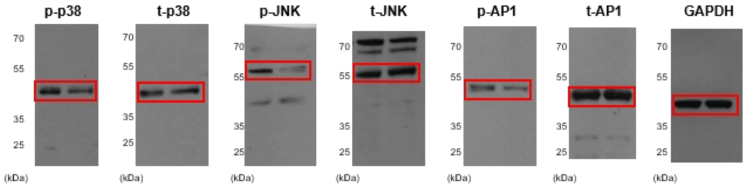

Fig 4h

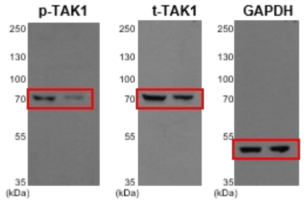

Fig 4j

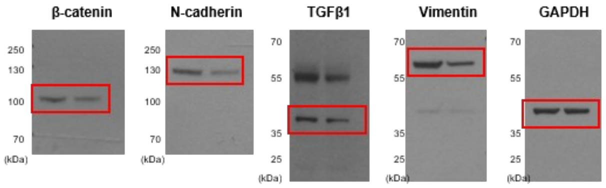

Fig 5d

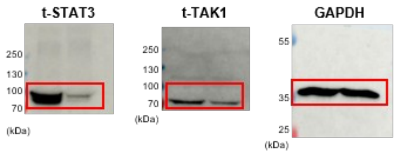

Fig 5a

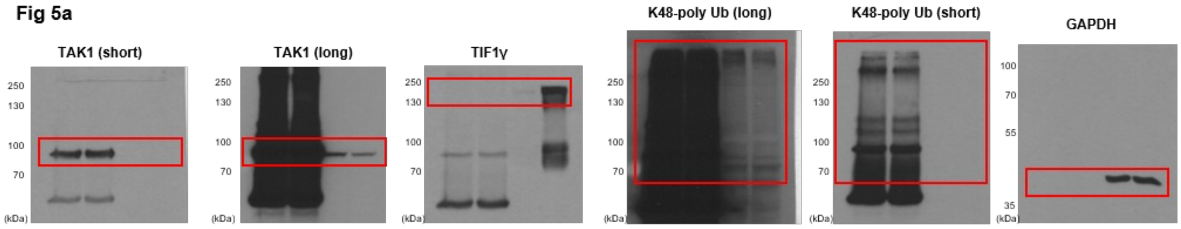

Fig 6a

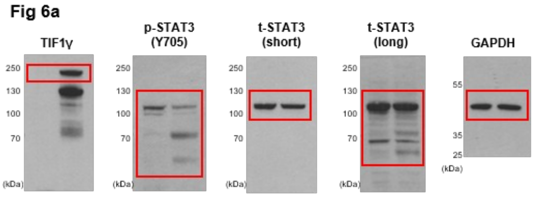

Fig 6c

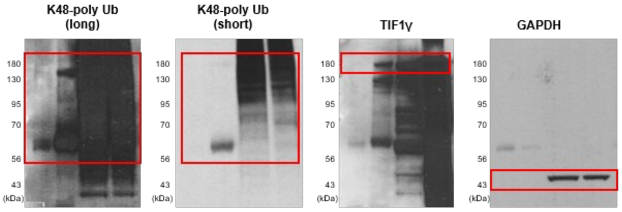

Fig 6d

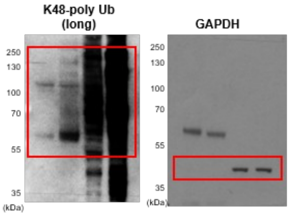

Fig 6e

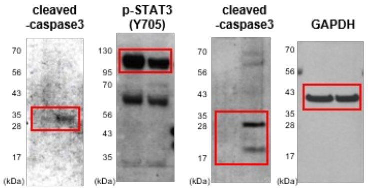

Fig 6f

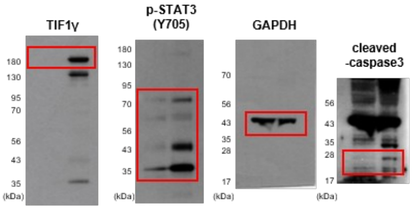

Fig 6h

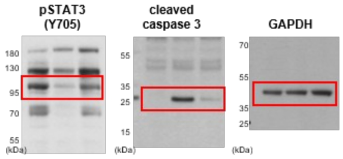

Fig 6g

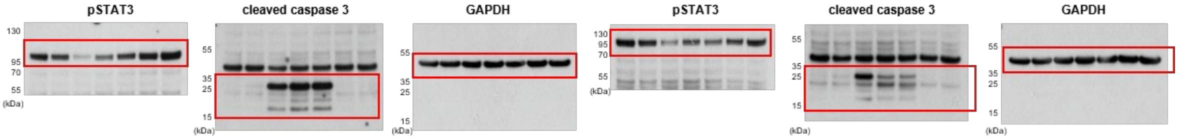

Fig 7f

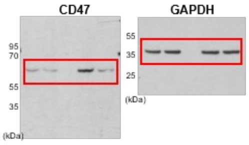

Fig 7h

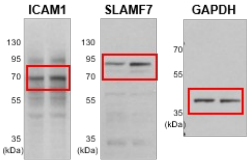

Fig S1b

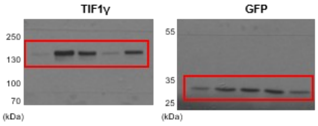

Fig S1e

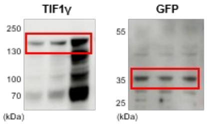

Fig S7b

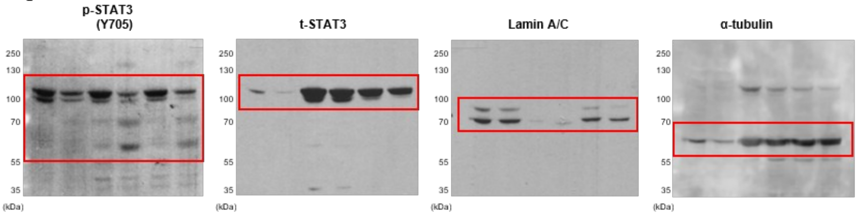

Fig S8b

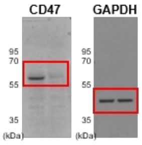

Fig S8l

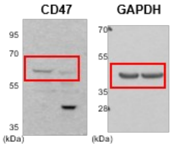

Fig S8o

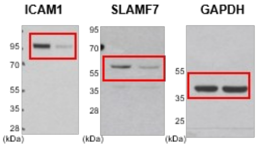

Supplement: Supplementary file 1 [file 41392_2026_2977_MOESM2_ESM.docx]
