# Supplementary material for: Transcriptional intermediary factor 1 gamma-based multitarget gene therapeutic strategy for triple-negative breast cancer
Source: Signal Transduct Target Ther. 2026 Sep 29;11:413. doi: 10.1038/s41392-026-02977-x (PMC13620165; doi:10.1038/s41392-026-02977-x)
Supplement: Supplementary file 1 [file 41392_2026_2977_MOESM1_ESM.docx]

Supplementary Materials for

**Transcriptional intermediary factor 1 gamma-based multitarget gene therapeutic strategy for triple-negative breast cancer**

Hyomin Park, Tae Yoon Kim, Hyunji Yun, Areum Cha, Dodam Moon, Eugene Yun, Youngmin Kim, Seock-Ah Im, Dae-Won Lee, Changhee Park, Hong-Kyu Kim, Han-Byoel Lee, Jaewon Lee, Nam-Joon Cho, Eun Ju Lee & Hyo-Soo Kim

Correspondence to: hyosoo@snu.ac.kr; [leeunju@snu.ac.kr](mailto:leeunju@snu.ac.kr)

**This PDF file includes:**

Materials and Methods

Figures S1 to S10

Table S1 to S7

**Other Supplementary Materials for this manuscript include the following:**

Videos S1 to S4

**Materials and Methods**

**Plasmid and siRNA transfection**

Plasmid DNA and siRNA transfections were performed using Lipofectamine 2000 (Cat. No. 116680019, Thermo Fisher Scientific) or Lipofectamine stem (Cat. No. STEM00015, Thermo Fisher Scientific) according to the manufacturer’s instructions. Predesigned siRNAs were purchased from Bioneer and diluted to a final concentration of 100 nM. The phMGFP plasmid (Cat. No. E6421, Promega) was used for the mock group. The plasmid encoding codon-optimized TIF1γ linked to a fluorescent reporter gene via an IRES element was synthesized by Macrogen. All plasmids were propagated in *Escherichia coli* (Cat. No. RH617, Real Biotech Corporation) and purified using an endotoxin-free plasmid DNA isolation kit (Cat. No. 740420.50, Macherey-Nagel) according to the manufacturer’s instructions. For adenoviral transduction experiments, recombinant adenoviruses encoding either codon-optimized TIF1γ or fluorescent reporters (GFP or tdTomato, for the mock controls) were transduced into cells at a multiplicity of infection (MOI) of 200.

**Coculture models**

For the 2-layer coculture model, Transwell inserts (six-well format) with 0.4 - or 1-µm pores (Cat. No. 3412, Costar; Cat. No. 353102, Falcon, respectively) were used to prevent cell migration across the membrane while allowing the exchange of soluble factors and potential interactions via tunneling nanotubes or other membrane-associated structures. For bottom-layer seeding, inserts were inverted and 700 µl of RPMI 1640 medium containing 100 nM PMA was applied evenly over the membrane. THP-1 monocytes were seeded onto the underside of the insert and incubated for 24 h to allow macrophage differentiation. Then, the medium was gently removed, and inserts were returned to the correct orientation in standard six-well plates containing fresh RPMI 1640 medium (10% FBS, 1% P/S). PDCs were seeded inside the upper chamber of the insert at a macrophage:cancer cell ratio of 4:1 and cultured for up to 48 h. Macrophages adhering to the lower surface of the insert were collected by gentle scraping and washed with phosphate-buffered saline (PBS). Cancer cells on the inner surface of the insert were collected separately using a scraper.

For the mixed coculture model, THP-1 monocytes were differentiated into macrophages through incubation with 100 nM PMA for 24 h. The macrophages were washed and seeded along with MDA-MB-231 cells at a macrophage:cancer cell ratio of 4:1. Cocultures were maintained up to 48 h in RPMI 1640 medium supplemented with 10% FBS and 1% P/S.

**Proteasome and caspase inhibition assay**

MDA-MB-231 cells were transfected with plasmid DNA and harvested 48 h after transfection. Inhibitor treatments were applied before harvest to match this endpoint. For caspase inhibition, Z-DEVD-FMK (Cat. No. HY-12466, MedChemExpress) and Z-LEHD-FMK (Cat. No. S7313, Selleck Chemicals) were dissolved in DMSO and added to the culture medium at a final concentration of 20 μM for the final 12 h before harvest. For proteasome inhibition, MG132 (Cat. No. M7449-200UL, Sigma-Aldrich) was added at final concentrations of 2.5 μM or 5 μM for the final 6 h before harvest. DMSO-treated cells were used as vehicle controls.

**Plasmid packaging for *in vivo* injection**

Liposome carriers were used for *in vivo* plasmid DNA delivery. Two types of liposomes were employed: Lipotrust (Cat. CSR-LEO10-EX, CosmoBio) and a custom-formulated liposome preparation referred to as “LipoDo.” Lipotrust was prepared according to the manufacturer’s instructions by reconstituting the lyophilized powder with UltraPure DNase/RNase-Free Distilled Water (Cat. 10977015, Gibco). When Lipotrust was unavailable, LipoDo was synthesized as follows. Three lipid components, i.e., DOTAP (Cat. 890890P, Avanti), DOPE (Cat. 850725P, Avanti), and cholesterol (Cat. 700000P, Avanti), were dissolved in *tert*-butanol (Cat. 471712, Sigma). The components were mixed at a molar ratio of 10 μmol (DOTAP):7.5 μmol (DOPE):7.5 μmol (cholesterol), and *tert*-butanol was added at four times the volume of the mixture. The mixture was frozen at –80 °C for at least 4 h and then lyophilized using a freeze dryer (Alpha 2-4 LSC; Martin Christ) for 2 days to produce a lipid cake. To rehydrate the lipid cake, 1 ml of ultrapure DNase/RNase-free distilled water was added, and the mixture was vortexed for 10 min to obtain a 10 mM stock solution (DOTAP standard). The mixture was further vortexed every 30 min in an ultrasonic water bath at 37 °C (Branson 5800) and sonicated for 2 h. It was incubated at 4 °C for at least 2 h before use.

For plasmid packaging, 18 µg of plasmid DNA was mixed with 140 nmol of liposome, vortexed briefly, and incubated at 25 °C for 20 min. The complex was concentrated using a Vivaspin2 concentrator (Cat. VS0252, VIVASCIENCE). Samples were centrifuged at 1,500 × *g*, 25 °C for 5 min three times. The upper chamber of the spin column was then inverted and centrifuged at 3,000 × *g*, 25 °C for 3 min. The final volume was adjusted to 20 µl using ultrapure DNase/RNase-free distilled water.

**MIND TNBC mouse model**

Female BALB/c nude mice (5 weeks old, 16–20 g, Orient Bio) were used in all experiments. The MIND TNBC injection model was established using a procedure adapted from published methods.^21^ MDA-MB-231 cells were transfected with plasmid DNA using Lipofectamine 2000 for 24 h, followed by detachment using 0.25% trypsin-EDTA. The cells were suspended in 0.04% trypan blue in PBS and counted. A total of 1 × 10^6^ transfected cells in 5 µL of 0.04% trypan blue in PBS were injected into the 4th or 7th nipple duct. Primary breast tumors were not formed in this model; lung metastases were the predominant lesion.

**Transcription factor candidate selection for TAK1**

To identify candidate transcription factors regulating TAK1 expression, we analyzed the 1-kb promoter region of MAP3K7 (TAK1) using PROMO (ALGGEN), Harmonizome, and MotifMap. STAT1 and ELK1 were selected based on their frequent appearance across these platforms. In addition, GeneCards (Qiagen TFBS dataset) identified STAT3 and SOX9 as top predicted transcription factor binding sites. Based on these analyses, four candidates (STAT1, STAT3, ELK1, and SOX9) were shortlisted for further evaluation.

**Nuclear/cytoplasmic fractionation**

Cells were collected by scraping and pelleted by centrifugation. The pellets were resuspended in three volumes of cytosol extraction buffer composed of 0.1% digitonin, 0.01% Triton X-100, protease inhibitor cocktail, and phosphatase inhibitor cocktail in PBS. After pipetting up and down 10 times to ensure proper lysis, the mixture was centrifuged at 3,500 × *g*, 4 °C for 5 min using a pre-cooled centrifuge. The supernatant was collected as the cytosolic protein fraction.

The remaining pellet was washed once with 1 ml of PBS followed by centrifugation at 3,500 × *g* at 4 °C for 5 min. The pellet was resuspended in RIPA buffer supplemented with protease and phosphatase inhibitor cocktails and incubated on ice for 10 min. After incubation, samples were centrifuged at 15,000×*g*, 4 °C for 15 min, and the supernatant was collected as the nuclear protein fraction.

To prepare total cell lysates (input), 10% of the total cell volume was set aside prior to cytosol extraction and directly lysed in RIPA buffer containing protease and phosphatase inhibitors.

All protein samples (cytosolic, nuclear, and total lysates) were subsequently analyzed by western blotting as described above.

**Immunohistochemistry**

Mouse tissues were fixed in 4% neutral-buffered formalin, embedded in paraffin, and sectioned into 4–5 µm sections. The sections were stained with H&E (Cat. No. ab24588, Abcam) following standard protocols.

**Transwell migration assay**

MDA-MB-231 cells were seeded in six-well plates (Cat. No. 353046, Falcon) at a density of 3 × 10^6^ cells per well. The cells were transfected with plasmid DNA or siRNA using standard protocols. After 24 h, the cells were washed with PBS, detached, and resuspended in serum-free medium. A total of 2.5 × 10^4^ cells in 200 µl of serum-free medium were added to the upper chamber of a 6.5-mm Transwell insert with an 8.0-µm pore polycarbonate membrane (Cat. No. 3422, Corning). The lower chamber contained 300 µl of complete growth medium as a chemoattractant. The cells were incubated at 37 °C in a humidified atmosphere with 5% CO_2_ for 16–20 h. After incubation, non-migrated cells on the upper side of the membrane were gently removed using a wet cotton swab. The migrated cells on the lower side were fixed with 4% paraformaldehyde at room temperature for 15 min and washed with PBS. The cells were stained with 0.02% crystal violet for 10 min and washed with PBS three times. Migrated cells were imaged using a microscope and quantified using the ImageJ software (National Institutes of Health).

**Cell viability assay**

MDA-MB-231 cells transfected with plasmid DNA or siRNA were harvested and seeded into three clear, flat-bottom, tissue culture-treated 96-well microplates (Cat. No. 3596, Corning) at 2–3 × 10^3^ cells per well. Each well contained 100 µl of complete growth medium. The cells were incubated in a humidified incubator at 37 °C with 5% CO_2_. To assess cell proliferation, one plate was collected at 24, 48, and 72 h post-seeding, 10 µl of Cell Counting Kit-8 (Cat. No. CK04, Dojindo) solution was added to each well, and the plate was incubated at 37 °C for 2 h. Then, the absorbance at 450 nm was measured using a microplate reader (VERSAmax, Molecular Devices). Absorbance values were normalized and plotted to evaluate relative cell proliferation over time.

**Promoter sequence retrieval and STAT3/SMAD-binding site analysis**

The promoter regions (–650/+10) of CD47, CXCL5, and MAP3K7 (TAK1) were obtained from the UCSC Genome Browser (hg38 assembly). The obtained promoter sequences were analyzed using the JASPAR database (https://jaspar.elixir.no/) with the vertebrate CORE collection to identify putative STAT3-binding sites and SMAD-binding elements.

**Generation of the TIF1γ RING domain mutant**

The pVAX1-CMV-opti-TIF1γ RING domain point mutant (C125A/C128A) was generated as previously described (DOI 10.1016/j.cell.2005.01.033). Site-directed mutagenesis was performed by Macrogen (Seoul, Republic of Korea), and the introduced mutations were confirmed by DNA sequencing.

**Quantitative reverse transcription PCR (RT-qPCR)**

Total RNA was isolated from cultured cells using TRIzol reagent (Cat. No. 15596026, Thermo Fisher Scientific) according to the manufacturer’s instructions. cDNA was synthesized from 1 µg of total RNA using a reverse transcription master mix (Cat. No. FSQ-201, Toyobo). qPCRs were run using FastStart Universal SYBR Green Master (Rox) (Cat. No. 04913914001, Roche) in a QuantStudio 5 Real-Time PCR System (Applied Biosystems). The peptidylprolyl isomerase A gene (PPIA) was used as an internal reference gene to normalize mRNA expression levels. All primers were custom-designed and synthesized at Macrogen. Relative gene expression was calculated using the ΔΔCt method. Primer sequences used for RT-qPCR are listed in Table S2.

**Western blot analysis**

Total protein was extracted from cells using RIPA lysis buffer (Cat. No. 89901, Thermo Fisher Scientific) supplemented with complete protease inhibitor cocktail (Cat. No. P3100-001, genDEPOT) and phosphatase inhibitor cocktail (Cat. No. P3200-001, genDEPOT) on ice for 15 min. The lysates were centrifuged at 15,000 ×*g*, 4 °C for 10 min. Total protein was quantified using the BCA assay (Cat. No. 23223, Thermo Fisher Scientific). Equal amounts of protein were separated using 10% SDS gel electrophoresis or 4%–12% Bis-Tris gradient gels (Cat. No. NP0321, Thermo Fisher Scientific), depending on the molecular weight of the target protein. For Bis-Tris gels, NuPAGE MOPS or MES SDS Running Buffer (Cat. Nos. NP0001 and NP0002) was used according to the manufacturer’s instructions. Proteins were transferred onto nitrocellulose or polyvinylidene difluoride membranes using an iBlot 2 gel transfer system (Cat. No. IB21001, Thermo Fisher Scientific). Membranes were blocked with 5% normal horse serum in PBS and incubated with primary antibodies at 4 °C overnight. After washing, the membranes were incubated with HRP-conjugated secondary antibodies (1:20,000; Jackson ImmunoResearch). Immunoreactive bands were visualized using enhanced chemiluminescent HRP substrate. Band intensities were quantified using ImageJ, and target protein levels were normalized to the corresponding loading control. Primary antibodies used for western blot analysis are listed in Table S3.

**Co-IP**

For co-IP experiments, MDA-MB-231 cells were seeded in 60-mm culture dishes and transfected with plasmid DNA expression vector for 48 h. Then, the cells were lysed in ice-cold lysis buffer containing 1% Triton X-100 and 0.1% SDS and supplemented with protease and phosphatase inhibitors on ice for 15 min. The lysates were centrifuged at 15,000 × g, 4 °C for 10 min to collect the supernatant. Protein concentrations were determined using the BCA assay. A portion of the lysate (5%) was reserved as input control. For IP, 2 µg of primary antibody was added to 1.5 mg of total protein and incubated on a rotator at 4 °C overnight. Immune complexes were captured via incubation with protein A/G agarose beads (Cat. No. ab196232, Abcam) at 4 °C under rotation for 2 h. Nonspecifically bound proteins were removed by washing with buffer containing 0.2% Tween-20. Subsequent immunoblotting was performed as described for western blot analysis. Primary antibodies used for co-IP are listed in Table S4.

**PLA**

The PLA was performed using the Duolink In Situ Red Starter Kit Mouse/Rabbit (Cat. No. DUO92101-1KT, Sigma-Aldrich) according to the manufacturer’s instructions. MDA-MB-231 cells were fixed with 4% paraformaldehyde for 15 min, permeabilized with 0.1% Triton X-100, and blocked using the supplied blocking solution. Cells were incubated with primary antibodies at 4 °C overnight, followed by incubation with PLA probes, ligation, and amplification to generate PLA signals. For dual PLA, an initial PLA reaction was completed and samples were washed and subjected to a second PLA reaction starting from primary antibody incubation. Fluorescence signals were acquired using a confocal microscope (Leica TCS SP8) and quantified using ImageJ software. Primary antibodies used for the PLA are listed in Table S5.

**Immunofluorescence**

Paraffin-embedded tissue sections were deparaffinized with xylene and rehydrated through a graded ethanol series, followed by heat-mediated antigen retrieval (Cat. No. ab93684, Abcam). OCT-embedded frozen tissue sections were washed in PBS to remove residual OCT compound, without antigen retrieval. For cell-based immunofluorescence, MDA-MB-231 cells were cultured on confocal-compatible dishes (µ-Dish 35 mm, Cat. No. 81156, Ibidi), washed with PBS, and fixed with 4% paraformaldehyde at room temperature for 15 min. Disseminated cells from *ex vivo* cultures were collected from the culture medium and centrifuged onto glass slides using a Cytospin 4 system (Thermo Scientific) at 800 rpm for 10 min, with single-use Cytofunnels (Cat. No. 59-910-40, Epredia).

All samples were blocked with 5% normal horse serum in PBS containing 0.01% Triton X-100 to reduce nonspecific binding and incubated with primary antibodies at 4 °C overnight. After washing with PBS, the samples were probed with Alexa Fluor-conjugated secondary antibodies (Invitrogen) at room temperature for 1.5 h. DAPI was used for nuclear counterstaining. Samples were washed with PBS containing 0.2% NP-40 and mounted using fluorescence mounting medium (Cat. No. S3023, DAKO). Fluorescence images were acquired using a TCS SP8 confocal microscope (Leica). Z-stack images were captured when appropriate, and confocal image acquisition and processing were performed using the LAS X software (Leica Application Suite X, Leica Microsystems). Fluorescence signals were quantified using ImageJ. Primary antibodies used for immunofluorescence staining are listed in Table S6.

**Chromatin immunoprecipitation**

MDA-MB-231 cells were transfected with plasmid DNA and harvested 24 h after transfection for chromatin immunoprecipitation analysis. For each immunoprecipitation reaction, 4 × 10⁶ cells were used. Cells were crosslinked with 1% formaldehyde at room temperature for 15 min. Subsequent procedures were performed using the Simple ChIP Enzymatic Chromatin IP Kit (Cat.No. 9003, CellSignalingTechnology) according to the manufacturer’s instructions. Chromatin was sheared using a Sonic Dismembrator Bioruptor (CosmoBio) at medium intensity with 4 cycles of 20 s sonication followed by 30 s resting intervals. Before antibody incubation, 2% of the chromatin input was removed and reserved as an input control. For immunoprecipitation, chromatin was incubated with 2 μg of total STAT3 antibody (Cat.No. 9139S, Cell Signaling Technology) overnight at 4 °C on a rotator. An equal amount of control IgG antibody provided in the kit was used as a negative control. Beads were then incubated with the samples at 4 °C for 2 h. After DNA purification, qPCR was performed as described in the RT-qPCR section of the Materials and Methods. Primer sets were designed to amplify the predicted STAT3-binding regions within the TAK1 promoter, for the negative control region and the three predicted STAT3-binding sites (SBSs) located at −536 to −523 (SBS #3), −284 to −271 (SBS #2), and −150 to −137 (SBS #1). ChIP enrichment at the predicted STAT3-binding regions within the TAK1 promoter was calculated as percent input using the following formula: Percent Input = 0.02 × 100 × 2*^(Ct Input−Ct IP)^*. Primer sequences used for ChIP-qPCR are listed in Table S7.

**TUNEL assay**

Paraffin-embedded tissue sections were deparaffinized with xylene and rehydrated through a graded alcohol series. Apoptotic cells were detected using the TUNEL Andy Fluor 647 Apoptosis Detection Kit (Cat. No. A052, ABP Biosciences) according to the manufacturer’s instructions. Fluorescence signals were imaged using a confocal microscope (Leica TCS SP8) and quantified using ImageJ.

**ELISA**

Culture supernatants were collected and filtered through a 0.45-µm syringe filter to remove cellular debris. CXCL5 protein levels in culture supernatants were measured using the Human CXCL5/ENA-78 Quantikine ELISA Kit (Cat. No. DX000, R&D Systems), according to the manufacturer’s instructions. Absorbance was measured using a microplate reader, and cytokine concentrations were calculated based on the standard curve provided with the kit. Because the endogenous secretion level of CXCL5 in MDA-MB-231 cells was low, cells were stimulated with TNFα (10 ng/mL) for 24 h prior to supernatant collection for ELISA analysis.

**Fig. S1 Construction and selection of the optimized TIF1γ gene therapy vector. a** Schematic diagram of codon optimization and plasmid structure of opti-hTIF1γ. The codon optimization strategy for opti-hTIF1γ was designed to improve translational efficiency in human cells by replacing rare codons with more frequently used human codons, increasing GC content, and removing inhibitory cis-acting motifs such as premature polyadenylation signals and cryptic splice sites. Illustration created with BioRender. **b,c** Western blot analysis of hTIF1γ expression in 293T cells transfected with terminally optimized constructs (N-term, C-term, N+C, or full-length); equal amounts of CMV-emGFP were co-transfected and used for normalization. Data represent the mean ± SD; n = 3. **d** Representative fluorescence images showing comparable transfection efficiency in MDA-MB-231 cells across all groups co-transfected with equal amounts of CMV-GFP plasmids. Scale bar, 100 μm. **e,f** Western blot analysis of hTIF1γ protein expression in MDA-MB-231 cells transfected with wild-type or codon-optimized TIF1γ constructs; CMV-emGFP served as an internal transfection control for normalization. Data represent the mean ± SD; n = 3.

**Fig. S2 Complete immunofluorescence dataset corresponding to Figure 1. a** Complete immunofluorescence images of patient-derived TNBC biopsy tissues (Patients 1–7) stained for the reporter gene, vimentin, Ki67, and DAPI following mock or opti-hTIF1γ transfection. Representative images are shown in Figure 1. Scale bars, 50 μm. **b** Complete TUNEL staining of patient-derived TNBC biopsy tissues (Patients 1–7) following mock or opti-hTIF1γ transfection. Representative images are shown in Figure 1. Scale bars, 50 μm.

**Fig. S3 Mouse TNBC ex vivo tumor culture model.** MDA-MB-231 cells were implanted into the mammary fat pads of mice to establish tumor masses. The excised tumors were sectioned and cultured using the same *ex vivo* culture procedure applied to patient-derived tumor tissues. Illustration created with BioRender.

**Fig. S4 Prevention of lung metastasis by opti-hTIF1γ in the MIND TNBC injection model in mice. a** Schematic illustration of the mammary intraductal (MIND) injection of wild or transfected TNBC cells (MDA-MB-231) with mock or opti-hTIF1γ plasmid. **b** Representative H&E staining images of lung tissues from mice injected with wild-type, mock-transduced, or opti-hTIF1γ-transduced TNBC cells. Scale bars, 400 μm. Quantification of H&E-defined metastatic area per 0.009 mm^2^ is shown on the right. Data represent the mean ± SD; n ≥ 9. **c** Immunofluorescence analysis of metastatic lung lesions from the mammary ductal injection of wild-type, mock-transduced, or opti-hTIF1γ-transduced TNBC cells. HLA-ABC (white) indicates human tumor cells; reporter genes are shown as GFP (green) or tdTomato (red); DAPI (blue). Scale bars, 20 μm. Quantification of HLA-ABC^+^ area among DAPI^+^ regions is shown on the right. Data represent the mean ± SD; n = 4. **d** Immunofluorescence images of lung tissues showing Ki67 (white) and DAPI (blue). The Ki67/DAPI ratio (%) represents proliferative cell density, and the DAPI^+^ area (%) represents metastatic burden per 0.33 mm^2^. Data represent the mean ± SD; n = 3. Scale bars, 50 μm.

**Fig. S5** **β-catenin and TGFβ signaling are required for proliferation and migration of TNBC cells. a** Cell Counting Kit-8 assay showing cell viability of MDA-MB-231 cells over 3 days after transfection with β-catenin or TGFβ1 siRNA. Absorbance at 450 nm was measured daily. Data represent the mean ± SD; n = 3. **b** Representative images of migrated MDA-MB-231 cells 2 days after siRNA transfection in a Transwell migration assay stained with crystal violet. Scale bar, 100 μm Quantification of the migrated area per 0.37 mm^2^ is shown on the right. Data represent the mean ± SD; n = 3. **c, d** Densitometric quantification of the western blot data shown in Fig. 4b and c, respectively. Data represent the mean ± SD; n = 3.

**Fig. S6** **Opti-hTIF1γ regulates STAT3-dependent signaling and TAK1 transcription in TNBC cells. a** Quantification of p-STAT3 and t-STAT3 from the western blot data shown in Fig. 6a. Protein levels were normalized to GAPDH. Data represent the mean ± SD; n = 4. **b** RT-qPCR analysis of hSTAT3 mRNA levels in MDA-MB-231 cells 24 and 48 h after opti-hTIF1γ transfection. Data represent the mean ± SD; n = 3. **c** Nuclear and cytosolic fractionation analysis of STAT3 signaling in MDA-MB-231 cells. **d** Quantification of p-STAT3 and cleaved caspase3 from the western blot data shown in Fig. 6g. Protein levels were normalized to GAPDH. Data represent the mean ± SD; n = 3. **e** Schematic summary of the opti-hTIF1γ-mediated inhibition of tumorigenic and metastatic signaling pathways in TNBC. Illustration created with BioRender.

**Fig. S7** **Opti-hTIF1γ suppresses CD47 and CXCL5 expression in TNBC cells and promotes macrophage activation. a** RT–qPCR analysis of human CD47 and CXCL5 expression in patient-derived cells (PDCs). Expression levels were normalized to human PPIA. Data represent the mean ± SEM; n = 3 independent experiments, with each data point representing an independent experiment measured at least in duplicate. **b** Western blot analysis of CD47 in PDCs and corresponding densitometric quantification. CD47 levels were normalized to GAPDH. Data represent the mean ± SD; n = 3. **c** Enzyme-linked immunosorbent assay (ELISA) of CXCL5 levels in PDC culture supernatants. Data represent the mean ± SD; n = 3 independent experiments, with each sample measured at least in duplicate. **d** Densitometric quantification of CD47 from the western blot data shown in Fig. 7c. CD47 levels were normalized to GAPDH. Data represent the mean ± SD; n = 3. **e** Densitometric quantification of ICAM1 and SLAMF7 in THP-1 macrophages from the western blot data shown in Fig. 7d. Protein levels were normalized to GAPDH. Data represent the mean ± SD; n = 3. **f** Schematic timeline of the mixed coculture experiment using MDA-MB-231 (TNBC) cells and PMA-induced THP-1 macrophages. **g** Immunofluorescence analysis of mock (tdTomato) or opti-hTIF1γ/tdTomato-transfected MDA-MB-231 cells cocultured with THP-1 macrophages. ICAM1 (green) marks activated macrophages, tdTomato (red) indicates transfected TNBC cells, and DAPI (blue) labels nuclei. Scale bars, 50 μm (top) and 20 μm (bottom). Quantification shows the percentage of tdTomato^+^ cells with normal morphology per 0.88 mm^2^. Mean ± SD, n ≥ 4. **h** Confocal Z-stack images of transfected MDA-MB-231 TNBC cells expressing tdTomato cocultured with ICAM1⁺ THP-1 macrophages. Scale bars, 10 μm. Phagocytosis was quantified as the percentage of tdTomato⁺ TNBC cells localized within ICAM1⁺ macrophages. Data represent the mean ± SD; n = 4. **i** RT–qPCR analysis of CD47 and CXCL5 expression in MDA-MB-231 cells. Expression levels were normalized to human PPIA. Data represent the mean ± SD; n = 3. **j** Western blot analysis of CD47 in MDA-MB-231 cells and corresponding quantification. CD47 levels were normalized to GAPDH. Data represent the mean ± SD; n = 3. **k** ELISA analysis of CXCL5 secretion from transfected MDA-MB-231 cells following TNF-α stimulation (10 ng/mL, 24 h). Data represent the mean ± SD; n = 4. **l** Immunofluorescence images of the mixed coculture showing CD47 (green) and tdTomato (red) expression in TNBC cells, with nuclei counterstained with DAPI (blue). Scale bars, 50 μm (top) and 20 μm (bottom).

**Fig. S8** **STAT3 transcriptionally regulates CD47 and CXCL5 expression in TNBC cells. a** Schematic representation of predicted STAT3-binding sites within the proximal promoter regions of the human CD47 and CXCL5 genes. **b** RT–qPCR analysis of CD47 and CXCL5 expression following siSTAT3-mediated knockdown in MDA-MB-231 cells. Expression levels were normalized to human PPIA. Data represent the mean ± SD; n = 3. **c** Western blot analysis of STAT3 and CD47 in MDA-MB-231 cells following siSTAT3-mediated knockdown, with corresponding densitometric quantification of CD47 normalized to GAPDH. Data represent the mean ± SD; n = 3. **d** ELISA analysis of CXCL5 secretion following siSTAT3-mediated knockdown in MDA-MB-231 cells stimulated with TNF-α (10 ng/mL). Data represent the mean ± SD; n = 3–4. **e** Schematic summary of opti-hTIF1γ-mediated suppression of STAT3-dependent CD47 and CXCL5 expression. Illustration created with BioRender.

**Fig. S9** **In silico promoter map identifying potential SMAD Binding Elements (SBEs) within human β-catenin, CD47 and CXCL5 proximal promoter regions. a, b** Schematic illustration depicting the in silico genomic screening and structural organization of the proximal promoter regions (-650 to -10 bp relative to the transcription start site, TSS) for β-catenin (a), human CD47 and CXCL5 genes (b).

**Fig. S10 Long-term chronic toxicity profiling and systemic safety evaluation of opti-hTIF1γ in mice.** **a** Schematic representation of the experimental timeline for the chronic safety study. Mice were subjected to long-term repeated intraperitoneal (I.P.) injections every 2 weeks for a total of 22 times over a 41-week period. **b** Representative histological images (H&E staining) of vital organs (from left to right: heart, brain, liver, lung, kidney, and spleen) isolated at the end of the 41-week regimen. No histopathological abnormalities, necrosis, or toxic lesions were observed across the treatment groups. Scale bar, 2 mm. **c** Serum biochemical analysis monitoring key physiological indicators of organ function. All tested panels demonstrated no statistically significant differences between the experimental cohorts, suggesting the absence of long-term systemic or off-target organ toxicities following repeated systemic exposure. Data represent the mean ± SD. n ≥ 4.

**Table S1.** Molecular characterization of the patient biopsies

| patient ID | gBRCA1/2 | p53 | Ki-67 | CK5/6 | vimentin | subtype-related feature |
| --- | --- | --- | --- | --- | --- | --- |
| #1 | not detected | > 75% | > 20% | < 10% | > 25% | mesenchymal-like |
| #2 | not detected | < 10% | > 40% | < 10% | > 25% | mesenchymal-like |
| #3 | not detected | > 75% | > 70% | < 10% | > 35% | mesenchymal-like |
| #4 | not detected | > 75% | > 60% | > 10% | > 20% | basal–mesenchymal |
| #5 | not detected | > 25% | > 50% | > 10% | > 25% | basal–mesenchymal |
| #6 | not detected | > 85% | > 25% | > 10% | > 20% | basal–mesenchymal |
| #7 | not detected | > 10% | > 90% | < 10% | > 20% | mesenchymal-like |

| patient ID | Clinical / pathologic Stage (T/N/M) | Histologic Grade | IHC results | | |
| --- | --- | --- | --- | --- | --- |
|  |  |  | ER | PR | HER2 |
| #1 | 3/2/0 | III/III | 1 (Negative) | 1 (Negative) | 1 (Negative) |
| #2 | 2/1/0 | III/III | 1 (Negative) | 1 (Negative) | 0 (Negative) |
| #3 | 2/1/0 | III/III | 1 (Negative) | 1 (Negative) | 0 (Negative) |
| #4 | 2/1/0 | III/III | 1 (Negative) | 1 (Negative) | 0 (Negative) |
| #5 | 2/0/0 | III/III | 1 (Negative) | 1 (Negative) | 1 (negative ; focal 2 <10%) |
| #6 | 3/1/0 | III/III | 1 (Negative) | 1 (Negative) | 1 (Negative) |
| #7 | 2/0/0 | III/III | 1 (Negative) | 1 (Negative) | 1 (negative ; focal 2 <10%) |
| ER/PR IHC : 0 = Positive; 1 = Negative HER2 IHC : 0 - 1 = Negative; 2 = Equivocal, 3 = Positive | | | | | |

**Table S2.** **Primer sequences used for RT-qPCR.**

| **Gene name** | **Primer** | **Sequence (5′→3′)** |
| --- | --- | --- |
| Human_PPIA | Forward | CATAATGGCACTGGTGGCAAG |
|  | Reverse | GCCATCCAACCACTCAGTCTT |
| Human_N-cadherin | Forward | GAGGCTTCTGGTGAAATCGCA |
|  | Reverse | CAGTTGCTAAACTTCACATTGAG |
| Human_CDH11 | Forward | TCCAATGGGTGAAGAAGAAGC |
|  | Reverse | ACTGTACGGTGGTCTTGCTG |
| Human_Vimentin | Forward | AAATGGCTCGTCACCTTCGT |
|  | Reverse | AGAAATCCTGCTCTCCTCGC |
| Human_β-catenin | Forward | GAGGAGCAGCTTCAGTCCC |
|  | Reverse | TCCAACTCCATCAAATCAGCTTG |
| Human_TGFβ1 | Forward | GGAAACCCACAACGAAATCTATG |
|  | Reverse | GAGAGCAACACGGGTTCAGG |
| Human_TAK1 | Forward | AGCAAGTTCCTGCCACAAATGATAC |
|  | Reverse | GCGGCGATCCTAGCTTCTATTTC |
| Human_STAT3 | Forward | CTGTGGGAAGAATCACGCCT |
|  | Reverse | ACATCCTGAAGGTGCTGCTC |
| Human_CD47 | Forward | TCGTGTTGTTTCATGGTTTTCTC |
|  | Reverse | TAGTCCAGCAACAAGTAAAGCAAT |
| Human_SLAMF7 | Forward | AGCAGCCAATGAGTCCCATAAT |
|  | Reverse | GTCATCAGCAGCACCTTCACAG |
| Human_CXCL5 | Forward | GCTGCGTTGCGTTTGTTTAC |
|  | Reverse | GTTCTTCAGGGAGGCTACCACT |
| Human_SIRPa | Forward | CAAATACCGCCGCTGAGAAC |
|  | Reverse | GGGCTTTCTTCTGTCTGATTCG |
| Human_ICAM1 | Forward | CCTATGGCAACGACTCCTTCTC |
|  | Reverse | CGGAAAGCTGTAGATGGTCACTG |

**Table S3.** **Primary antibodies used for western blot analysis.**

| **Target protein** | **Vendor** | **Cat. No.** | **Dilution factor** |
| --- | --- | --- | --- |
| TIF1γ | Abcam | ab47062 | 1:1,000 |
| N-cadherin | BD Biosciences | 610920 | 1:1,000 |
| Vimentin | Cell Signaling Technology | 5741s | 1:1,000 |
| CDH11 | Abcam | ab151302 | 1:1,000 |
| c-Myc | Cell Signaling Technology | 2276s | 1:1,000 |
| Cyclin D1 | Cell Signaling Technology | 2922s | 1:1,000 |
| BCL-2 | ABclonal | A19693 | 1:1,000 |
| BAX | Cell Signaling Technology | 2772s | 1:1,000 |
| pro-caspase-3 | Cell Signaling Technology | 9662s | 1:1,000 |
| cleaved caspase-3 | Cell Signaling Technology | 9661s | 1:1,000 |
| TGFβ1 | Abcam | ab92486 | 1:1,000 |
| β-catenin | Santa Cruz biotechnology | Sc-sc7199 | 1:1,000 |
| p-p38 | Cell Signaling Technology | 4511s | 1:1,000 |
| t-p38 | Cell Signaling Technology | 8690s | 1:1,000 |
| p-JNK | Cell Signaling Technology | 4668s | 1:1,000 |
| t-JNK | Cell Signaling Technology | 9252s | 1:1,000 |
| p-AP1 | Cell Signaling Technology | 9261s | 1:1,000 |
| t-AP1 | Cell Signaling Technology | 9165s | 1:1,000 |
| p-TAK1 | Cell Signaling Technology | 9339s | 1:1,000 |
| t-TAK1 | Cell Signaling Technology | 5206s | 1:1,000 |
| p-STAT3 (Y705) | Cell Signaling Technology | 9145s | 1:1,000 |
| t-STAT3 | Cell Signaling Technology | 9139s | 1:1,000 |
| Lamin A/C | Cell Signaling Technology | 2032s | 1:1,000 |
| α-Tubulin | Sigma-Aldrich | T6074 | 1:1,000 |
| K48-Poly Ub | Cell Signaling Technology | 8081s | 1:1,000 |
| ICAM1 | Abcam | ab2213 | 1:1,000 |
| CD47 | eBioscience | 14-0479-82 | 1:1,000 |
| SLAMF7 | ABclonal | A5782 | 1:1,000 |
| GFP | Thermo Fisher Scientific | A6455 | 1:1,000 |
| GAPDH | Thermo Fisher Scientific | MA5-15738 | 1:3,000 |
| β-actin | Santa Cruz biotechnology | sc-47778 | 1:3,000 |

**Table S4.** **Primary antibodies used for co-immunoprecipitation (Co-IP) assays.**

| **Protein** | **Vender** | **Cat. No.** |
| --- | --- | --- |
| β-catenin | Santa Cruz Biotechnology | sc-7963 |
| TAK1 | Cell Signaling Technology | 5206s |
| p-STAT3 (Y705) | Cell Signaling Technology | 9145s |
| p-STAT3 (Y705) | Thermo Fisher Scientific | MA5-15193 |
| t-STAT3 | Cell Signaling Technology | 9139s |
| cleaved caspase-3 | Proteintech | 68773-1 |

**Table S5.** **Primary antibodies used for proximity ligation assay (PLA).**

| **Protein** | **Vendor** | **Cat. No.** | **Dilution factor** |
| --- | --- | --- | --- |
| TIF1γ | Abcam | ab47062 | 1:50 |
| SMAD2 | Invitrogen | MA5-15877 | 1:50 |
| cleaved caspase-3 | Cell Signaling Technology | 9661s | 1:50 |
| STAT3 | Invitrogen | PA1-9105 | 1:50 |

**Table S6.** **Primary antibodies used for immunofluorescence (IF) staining.**

| **Protein** | **Vendor** | **Cat. No.** | **Dilution factor** |
| --- | --- | --- | --- |
| Vimentin | R&D Systems | MAB2105 | 1:100 |
| Ki67 | Abcam | ab89901 | 1:100 |
| CD68 | Santa Cruz | sc-70761 | 1:50 |
| ICAM1 | Abcam | ab2213 | 1:50 |
| HLA-ABC | Abcam | ab134189 | 1:50 |
| CD47 | Thermo Fisher Scientific | 14-0479-82 | 1:50 |

**Table S7.** **Primer sequences used for ChIP-qPCR.**

| **Gene name** | **Primer** | **Sequence (5′→3′)** |
| --- | --- | --- |
| TAK1 Neg SBS | Forward | GTGGTGGGCATATAGGTGTTCA |
|  | Reverse | CACAGCCATGATGCAGTTCTC |
| TAK1 SBS #3 | Forward | GAATTTCTCATAGCGTCAGGTACTA |
|  | Reverse | TTCATCCCTAAACTAAGTTGACCTG |
| TAK1 SBS #2 | Forward | TTTATCTTTCCCGCTGGTCC |
|  | Reverse | CGAGAGAGGACGCCTGACG |
| TAK1 SBS #1 | Forward | CGAAATTTGTTCTAGAGGTCCAAG |
|  | Reverse | GGAAAGAAAAGGAGGGACAGG |

*SBS : predicted STAT3 binding site

**Video S1.** **Live imaging of mock-transfected TNBC cells.**

MDA-MB-231 cells were transfected with mock (tdTomato) plasmid and monitored using live fluorescence imaging for 24–56 h after transfection. Images were captured every 10 min. The yellow box indicates the representative field shown in Fig. 3a.

**Video S2.** **Live imaging of opti-hTIF1γ–transfected TNBC cells.**

MDA-MB-231 cells were transfected with opti-hTIF1γ-IRES-tdTomato plasmid and monitored by live fluorescence imaging for 24–56 h after transfection. Images were captured every 10 min. The yellow box indicates the representative field shown in Fig. 3a.

**Video S3.** **Live imaging of mock-transfected TNBC cells cocultured with macrophages.**

THP-1 monocytes were differentiated into M0 macrophages by stimulation with phorbol 12-myristate 13-acetate (PMA) for 24 h and stained with DiO (green) for 15 min. MDA-MB-231 TNBC cells (mock, tdTomato; red) were transfected for 48 h and then mixed with THP-1–derived M0 macrophages for coculture. Live fluorescence imaging was performed every 15 min for 36 h. The yellow box indicates the representative field shown in Fig. 7g.

**Video S4.** **Live imaging of opti-hTIF1γ–transfected TNBC cells cocultured with macrophages.**

THP-1 monocytes were differentiated into M0 macrophages by stimulation with PMA for 24 h and stained with DiO (green) for 15 min. MDA-MB-231 TNBC cells (opti-hTIF1γ-tdTomato; red) were transfected for 48 h and then mixed with THP-1–derived M0 macrophages for coculture. Live fluorescence imaging was performed every 15 min for 36 h. The yellow box indicates the representative field shown in Fig. 7g.
